# Supplementary material for: Immune microenvironment remodeling after radiation of a progressing brain metastasis
Source: Cell Rep Med. 2023 May 19;4(6):101054. doi: 10.1016/j.xcrm.2023.101054 (PMC10313918; doi:10.1016/j.xcrm.2023.101054)
Supplement: Document S1. Figures S1–S6 [file mmc1.pdf]

Cell Reports Medicine, Volume *4*

Supplemental information

Immune microenvironment remodeling after radiation of a progressing brain metastasis

William H. Hudson, Jeffrey J. Olson, and Lisa J. Sudmeier

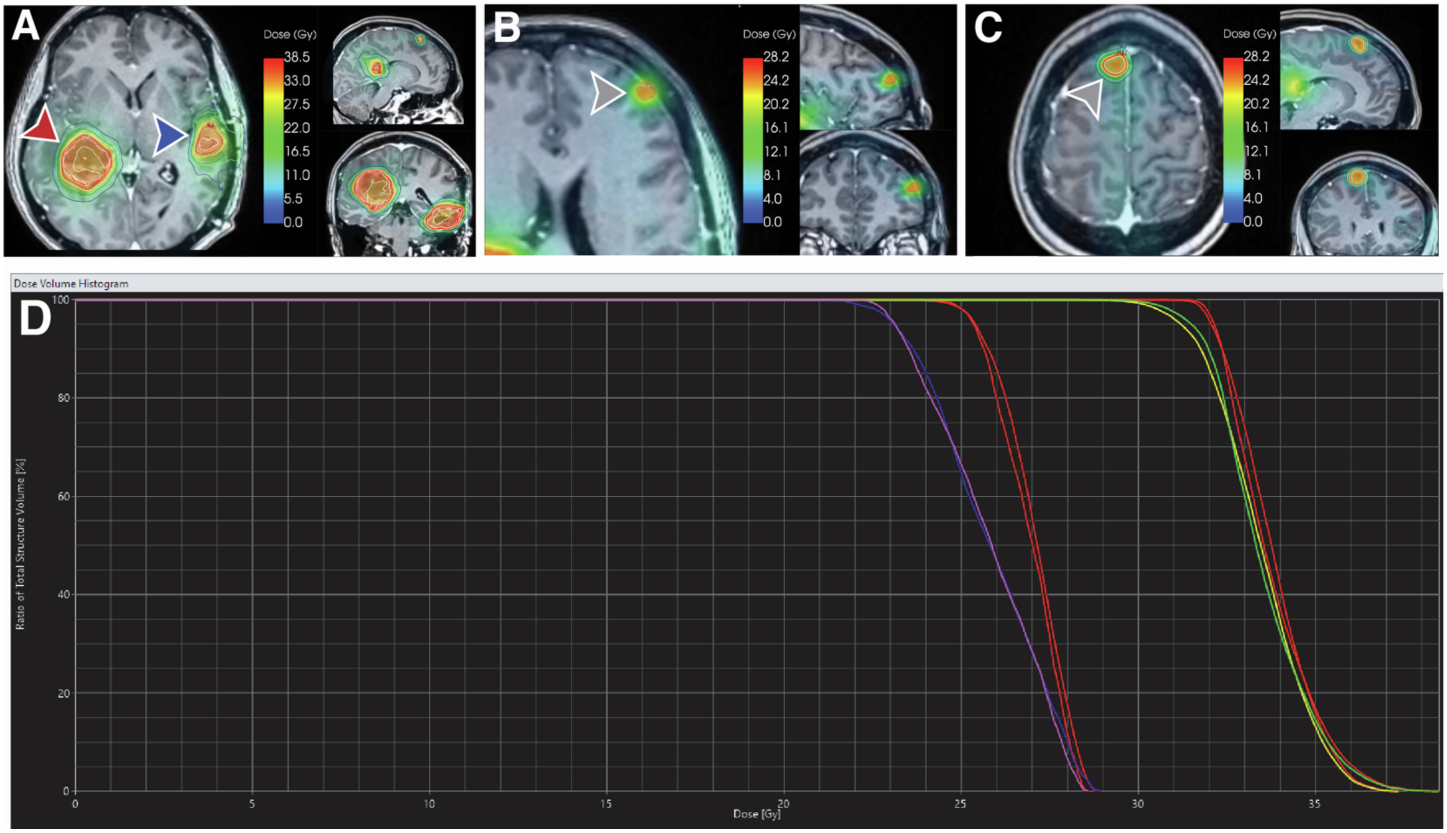

Figure S1, related to Figure 1: Stereotactic radiosurgery plans for the 4 treated lesions. **A)** The left temporal resection cavity (blue) and right frontoparietal lesion (red) that ultimately progressed were each treated to 30 Gy in 5 fractions of 6 Gy each. The unresected left frontal **(B)** and right frontal **(C)** tumors were each treated to 21 Gy in a single fraction. **D)** Dose volume showing coverage of the targets. Red lines show gross tumor volumes (GTVs) for each tumor. The GTV encompasses the entire contrast-enhancing tumor visualized on T1 post-contrast MRI. Red lines: Gross Tumor Volumes (GTV) for each tumor. Blue, Purple, Yellow, and Green lines: Planning Target Volume (PTV) for each tumor. The PTVs were generated by expanding the GTV 1mm isometrically, which the exception of the Left temporal lobe resection cavity GTV, which was expanded by 2mm to generate the PTV. Blue lines: Left Frontal PTV (shown in B); purple lines: Right Frontal PTV (shown in C); yellow lines: Left temporal resection cavity PTV (shown in A); green lines: right frontoparietal PTV (also shown in A)

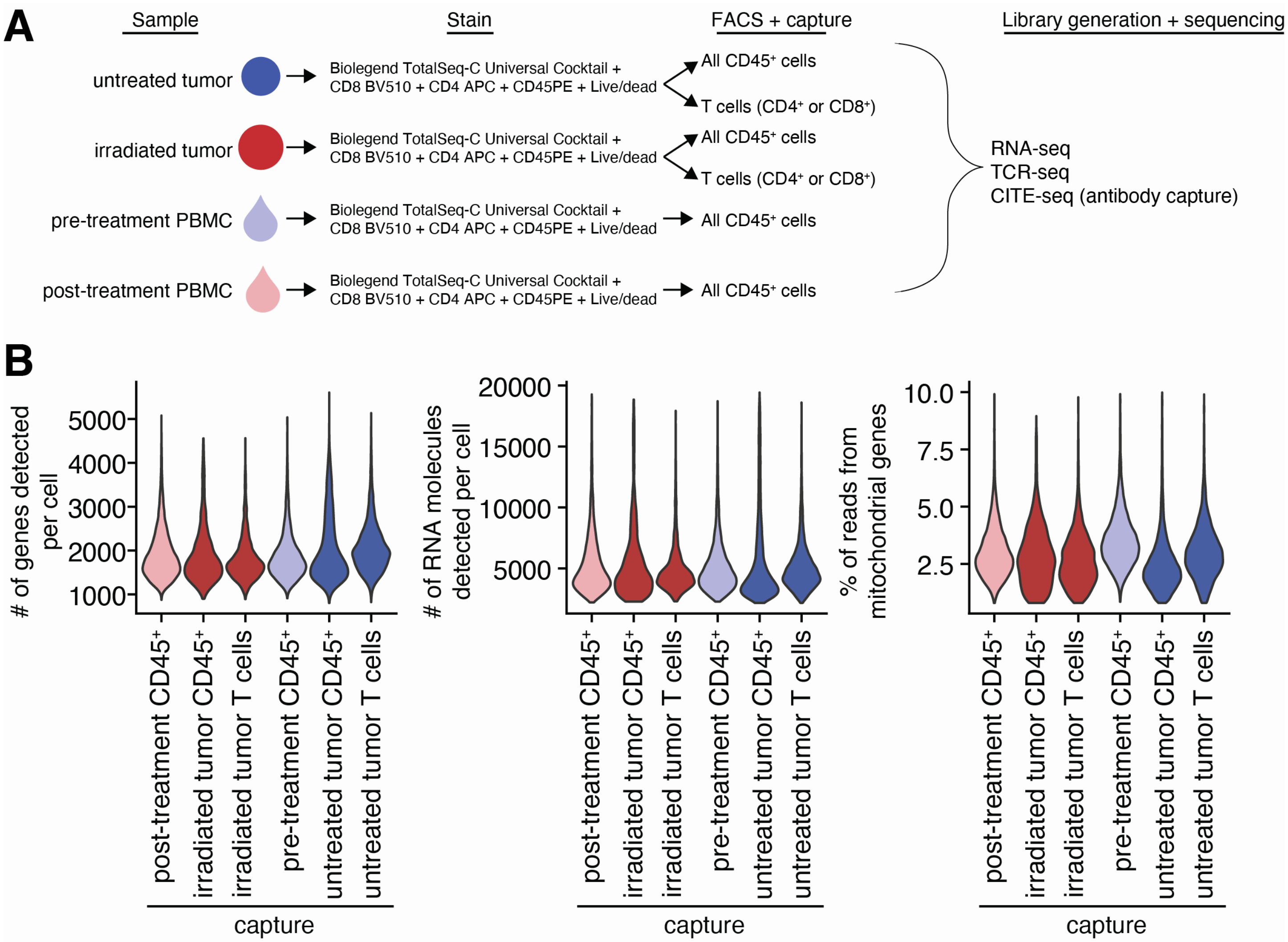

Figure S2, related to Figure 2: Single-cell experiments performed on samples described in this study. A) All four samples were stained with the Biolegend Totalseq-C Universal Cocktail, a mixture of antibodies against 130 unique surface antigens. Cells were additionally stained with fluorescently-labeled, non-competing clones against CD4, CD8, and CD45 for flow sorting. Live $CD45^+$ cells were sorted from all samples, and an additional pool of T cells ($CD45^+$ and $CD4^+$ or $CD8^+$) were sorted from the two tumor samples. RNA-sequencing, TCR-sequencing, and CITE-sequencing (antibody capture) libraries were generated and sequenced from all sorted populations. **B)** Quality control metrics of gene expression libraries from all six samples.

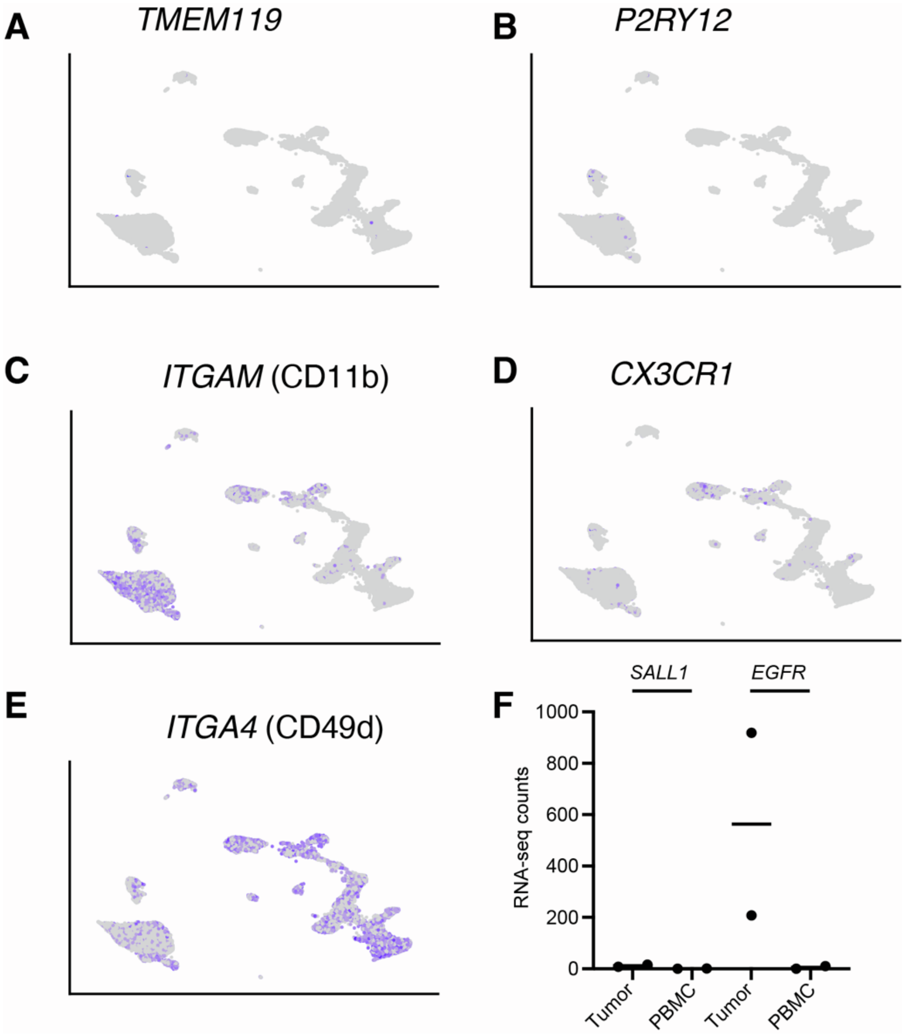

Figure S3, related to Figure 3: Limited expression of microglia-associated genes within the tumor. A-E) Expression of selected myeloid markers in single-cell RNA-sequencing data set. A and B indicate limited expression of microglia markers *TMEM119* and *P2RY12* genes among tumor-infiltrating immune cells. F) The microglia marker gene *SALL1* was not detected in single-cell RNA-sequencing data, and bulk RNA-sequencing data is shown instead. The lung tumor tissue marker gene *EGFR* is shown for comparison.

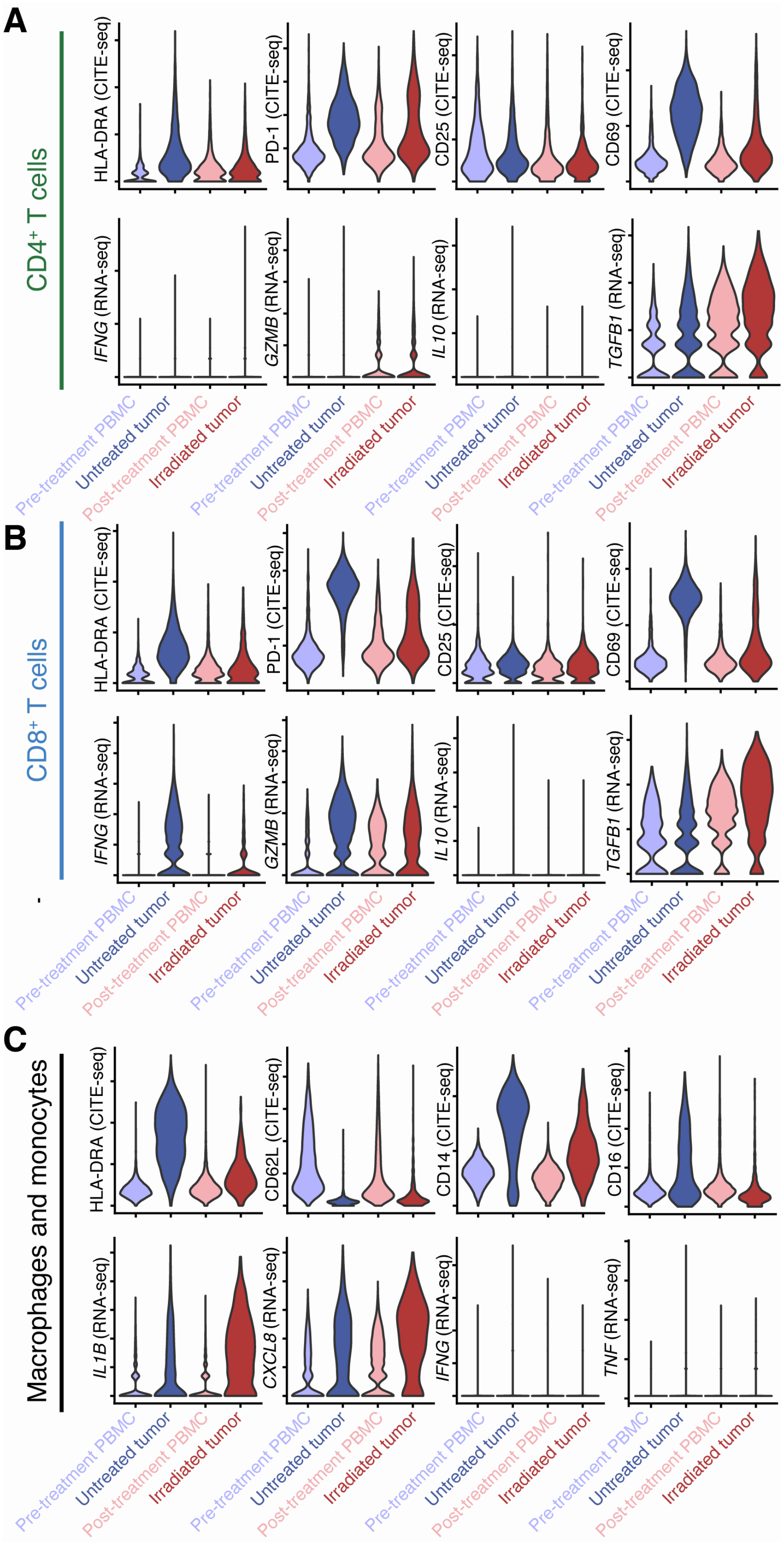

Figure S4, related to Figure 4: Expression of selected markers associated with activation and functional status of CD4$^+$ T cells (A), CD8$^+$ T cells (B), and macrophages/monocytes (C).

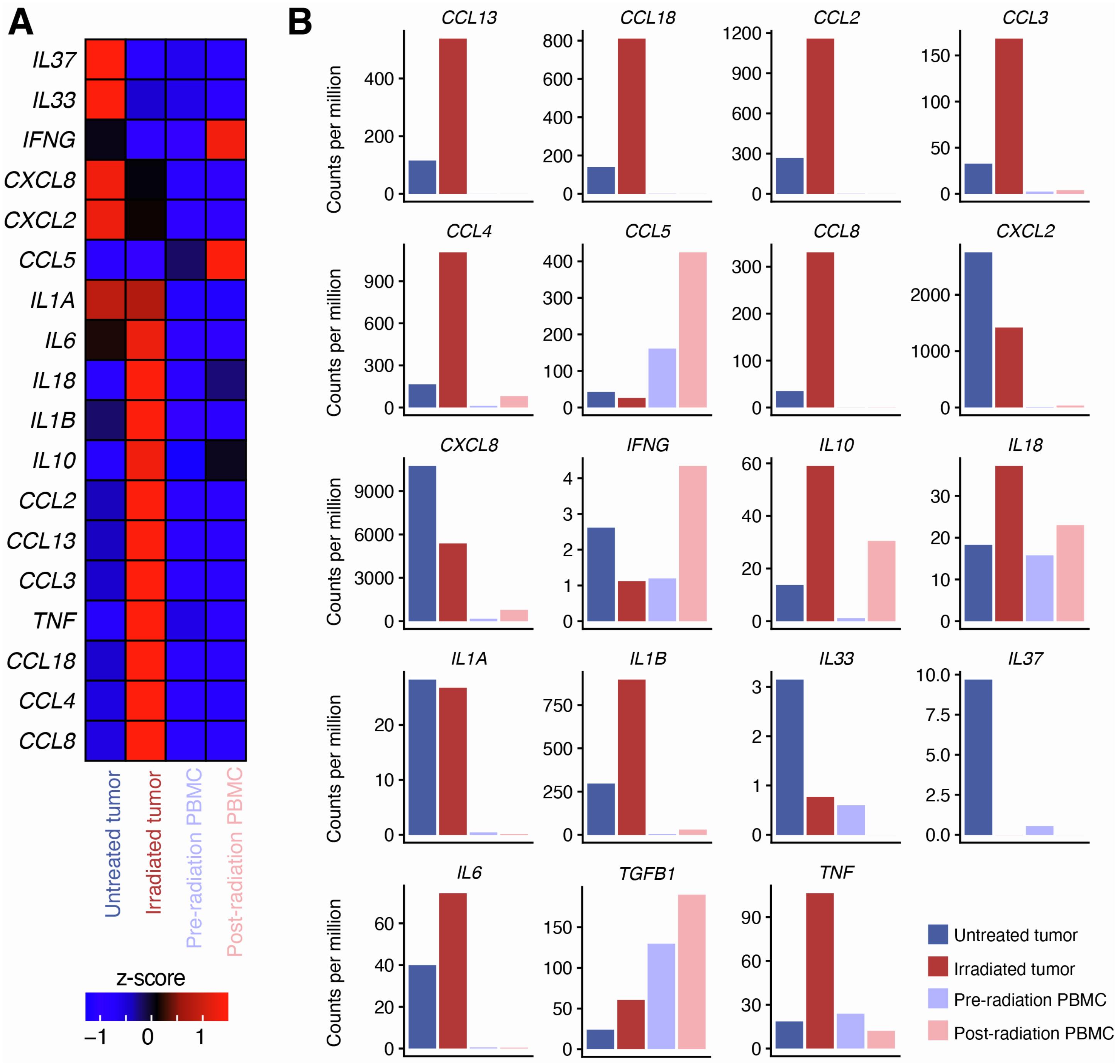

Figure S5, related to Figure 5: Relative (A) and absolute (B) expression of cytokine genes in PBMC and lymphocyte-depleted tumor samples.

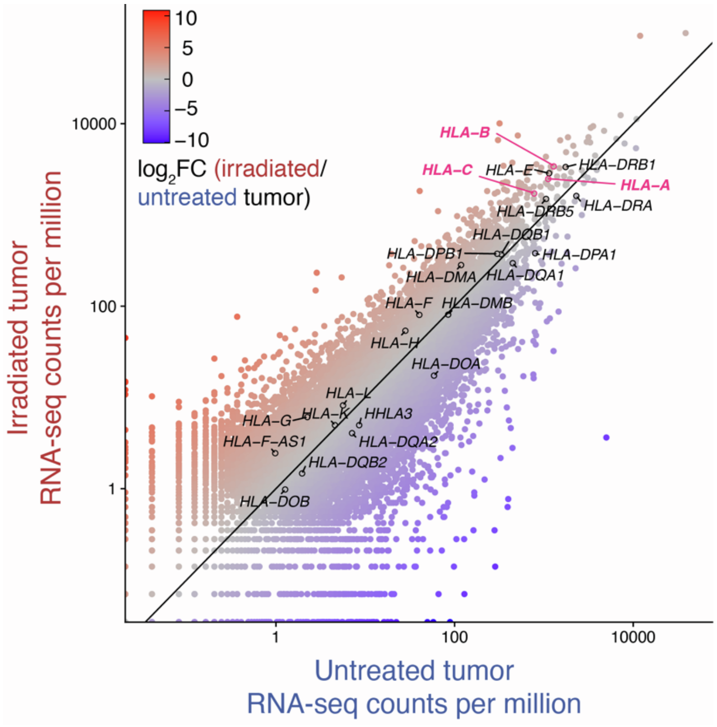

Figure S6, related to Figure 5: mRNA expression of HLA genes in the untreated (x-axis) and irradiated tumor (y-axis). HLA transcript expression was well correlated (r=0.84). Classical MHC I genes (HLA-A, -B, and -C) were among the highest expressed genes in both tumors and are shown labeled in pink.
