# Supplementary material for: Immune microenvironment remodeling after radiation of a progressing brain metastasis
Source: Cell Rep Med. 2023 May 19;4(6):101054. doi: 10.1016/j.xcrm.2023.101054 (PMC10313918; doi:10.1016/j.xcrm.2023.101054)
Supplement: Document S2. Article plus supplemental information [file mmc5.pdf]

Cell Reports
Medicine

Report

Immune microenvironment remodeling after radiation of a progressing brain metastasis

Graphical abstract

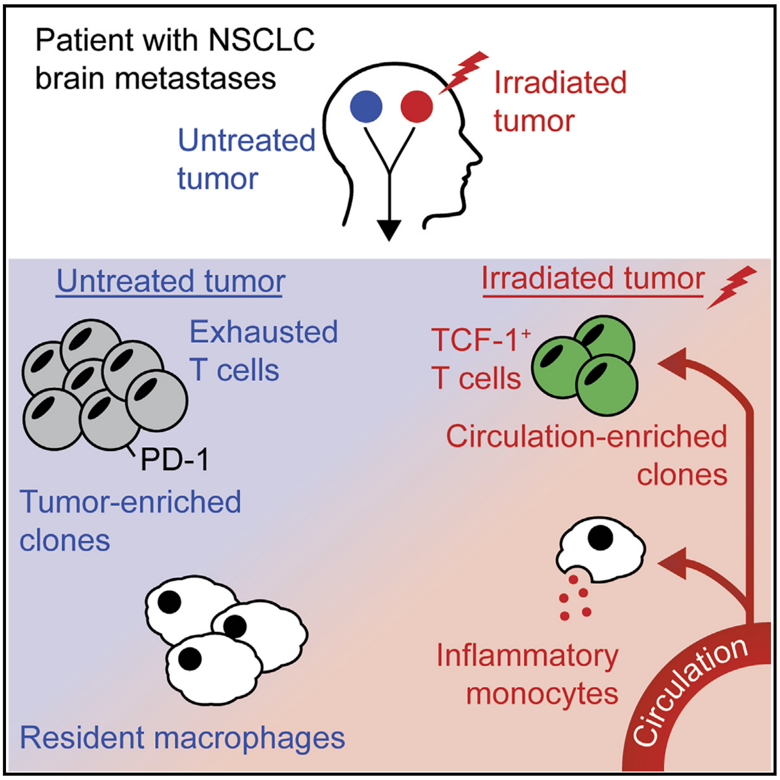

Highlights

- Detailed analysis of a matched pair of untreated and irradiated NSCLC metastases
- Radiation is associated with reduced overall immune infiltration
- Pro-inflammatory monocytes infiltrate the treated tumor
- Tumor-enriched T cell clones are depleted in the irradiated lesion

Authors

William H. Hudson, Jeffrey J. Olson, Lisa J. Sudmeier

Correspondence

william.hudson@bcm.edu (W.H.H.),
lisa.jane.sudmeier@emory.edu (L.J.S.)

In brief

The effects of radiation on the tumor immune microenvironment are unclear. Hudson et al. report a detailed characterization of an autologous pair of non-small cell lung cancer brain metastases. Radiation is associated with extensive immune remodeling, including infiltration of inflammatory monocytes and depletion of tumor-enriched $CD8^+$ T cells.

Hudson et al., 2023, Cell Reports Medicine *4*, 101054
June 20, 2023 © 2023 The Author(s).
https://doi.org/10.1016/j.xcrm.2023.101054

CellPress

Cell Reports Medicine

CellPress
OPEN ACCESS

Report

Immune microenvironment remodeling after radiation of a progressing brain metastasis

William H. Hudson,[1,2,3,*] Jeffrey J. Olson,[4,5] and Lisa J. Sudmeier[5,6,7,*]
[1]Department of Molecular and Cellular Biology, Baylor College of Medicine, Houston, TX 77030, USA
[2]Center for Cell and Gene Therapy, Baylor College of Medicine, Houston, TX 77030, USA
[3]Dan L. Duncan Comprehensive Cancer Center, Baylor College of Medicine, Houston, TX 77030, USA
[4]Department of Neurological Surgery, Emory University School of Medicine, Atlanta, GA 30322, USA
[5]Winship Cancer Institute, Emory University School of Medicine, Atlanta, GA 30322, USA
[6]Department of Radiation Oncology, Emory University School of Medicine, Atlanta, GA 30322, USA
[7]Lead contact
*Correspondence: william.hudson@bcm.edu (W.H.H.), lisa.jane.sudmeier@emory.edu (L.J.S.)
https://doi.org/10.1016/j.xcrm.2023.101054

SUMMARY

Radiation is commonly used in the treatment of many cancers. However, its effects on anti-tumor immune responses are incompletely understood. Here, we present a detailed immunological analysis of two tumors from a patient with multiple non-small cell lung cancer metastases to the brain. One tumor was resected without treatment; the second was irradiated to a total dose of 30 Gy and resected following further progression. Comprehensive single-cell analysis reveals a substantially reduced immune cell fraction in the irradiated tumor, including the depletion of tissue-resident macrophages and infiltration of pro-inflammatory monocytes. Despite the presence of similar somatic mutations in both tumors, radiation is associated with the depletion of exhausted, tumor-resident T cell clones and their replacement by circulating clones unlikely to contribute to tumor-specific immunity. These results provide insight into the local effects of radiation on anti-tumor immunity and raise important considerations for the combination of radiation and immunotherapy.

INTRODUCTION

Promoting and generating anti-tumor immunity is an emerging and important strategy to treat cancer. Immune checkpoint blockade (ICB), which restricts the function of inhibitory signaling molecules expressed by exhausted T cells, is now a mainstay in the treatment of many cancer types.[1] However, the poor response of many patients to ICB has spurred the search for combination therapies to improve their efficacy. In many cases, this involves targeting multiple immune checkpoint receptors present on T cells, such as PD-1 and CTLA-4.[2] Another active area of investigation is the combination of ICB with radiation therapy.[3]

Radiation has been employed to directly kill cancer cells for over a century. With the recent advent of cancer immunotherapy, radiation has also been studied for the ability to promote anti-tumor immunity and thus synergize with ICB.[4,5] However, the differential effects of radiation and ICB within the tumor microenvironment (TME) pose a paradox for such strategies[6]: immune cells—particularly lymphocytes—are highly sensitive to death by radiation, yet immunotherapies rely on the survival and expansion of these same cells.[7,8] Thus, in order to design optimal therapies combining ICB and radiation, it is critical to determine the effects of radiation on tumor-specific T cell responses.

Here, we report a study of two brain metastases resected from a single patient with non-small cell lung cancer (NSCLC). One tumor was resected without treatment, and the second tumor was resected following stereotactic radiosurgery (SRS), which delivers a high dose of conformal radiation to the tumor or surgical resection cavity.[9] We show that, despite similar mutational profiles between the two tumors, radiation therapy is associated with dramatic changes in the tumor immune microenvironment. The irradiated tumor was infiltrated by fewer immune cells in total, with higher frequencies of interleukin 1β (IL-1β)-expressing monocytes. T cell infiltration was dramatically lower in the irradiated sample. Because these are autologous samples, we can directly compare immune receptor sequences between the two tumors, providing insight into the antigen specificity of T cells in the untreated and irradiated tumors. While the T cells in the irradiated tumor had a more functional phenotype, radiation was associated with the depletion of tumor-enriched clones and increased infiltration of circulating clones, indicating the likely ablation of tumor-specific T cells and their replacement by circulating bystander clones.

RESULTS

Case history

A patient with NSCLC was treated at Emory University Hospital for multiple brain metastases. One large left temporal tumor was surgically resected without additional therapy (termed the untreated tumor sample), and SRS to the resection cavity and three other tumors was performed 21–27 days post-surgery (Figure 1A). The resection cavity and a right frontoparietal lobe metastasis

© 2023 The Author(s).
This is an open access article under the CC BY license (http://creativecommons.org/licenses/by/4.0/).

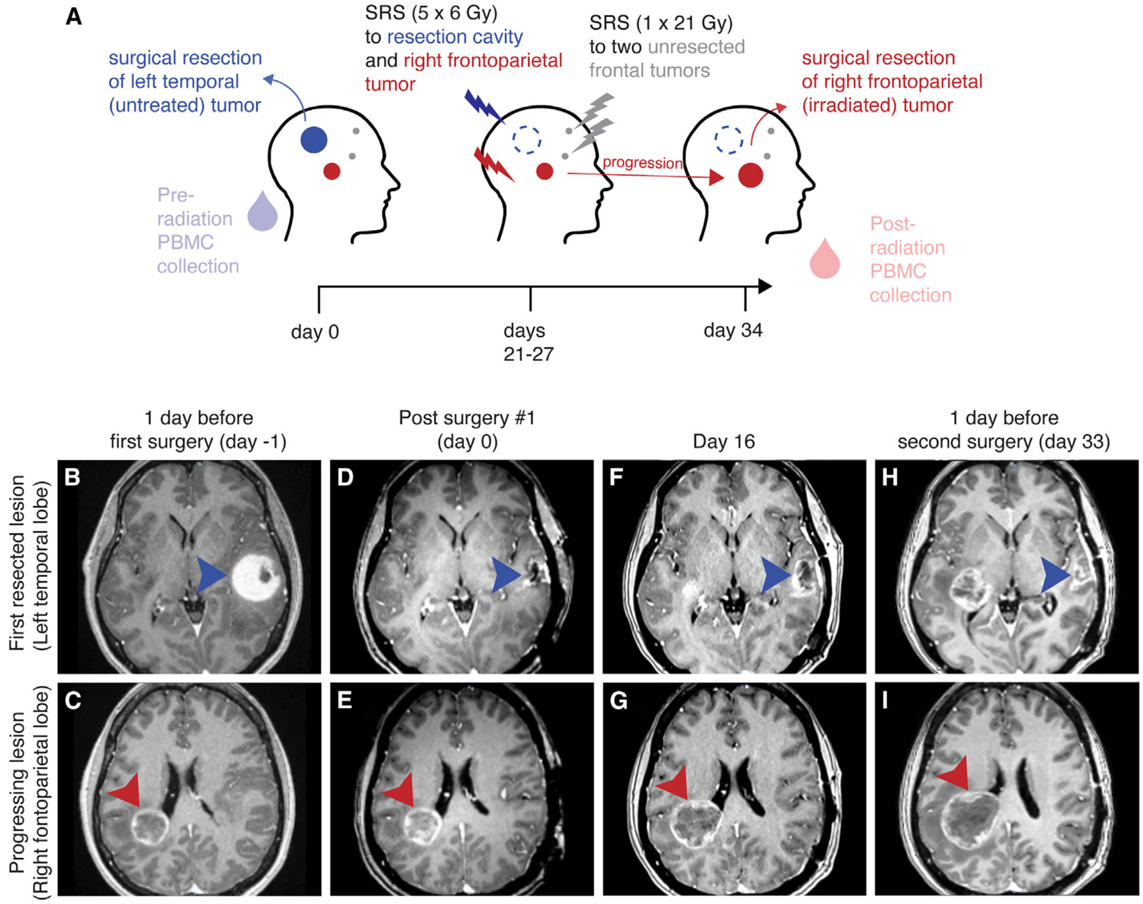

Figure 1. Case history of a patient with two resected brain metastases

(A) A patient presented with four NSCLC metastases. A large left temporal tumor was surgically resected. The resection cavity (dark blue) and a right frontoparietal lesion (dark red) were treated with 5 × 6 Gy stereotactic radiosurgery (SRS). Two smaller frontal lesions were treated with 1 × 21 Gy. The right frontoparietal tumor progressed throughout treatment and was resected 34 days after the initial surgery. Blood was collected and PBMCs isolated at the time of the initial (pre-radiation) and second (post-radiation) surgeries.

(B–I) T1 post-contrast magnetic resonance imaging (MRI) throughout the course of treatment. Arrowheads point to the untreated (dark blue) or irradiated (dark red) tumors.

(diameter 3 cm) each received 30 Gy in 5 fractions of 6 Gy each (Figure S1A). The two other lesions, one in the right frontal lobe and the other in the left frontal lobe, each measuring 0.5 cm in diameter, were each treated to 21 Gy in a single fraction (Figures S1B–S1D).

The right frontoparietal tumor progressed through radiation and was surgically resected 34 days after the first operation (termed the irradiated tumor sample). The patient was prescribed dexamethasone (10 mg bolus +4 mg/6 h) 2 days prior to the first surgery, tapered, and restarted with an identical regimen 3 days prior to the second surgery. No other systemic treatment was administered. Tumor and peripheral blood mononuclear cells (PBMCs) were collected at the time of each surgery (termed the pre-radiation PBMCs and post-radiation PBMCs, respectively).

Lower immune infiltration in the irradiated tumor

We quantified the immune infiltrate of each tumor by flow cytometry: the untreated tumor was infiltrated by 1.9×10^6 lymphocytes and 1×10^6 $CD8^+$ T cells per gram of tissue; the irradiated tumor was infiltrated by 2.7×10^5 and 6.1×10^4 lymphocytes and $CD8^+$ T cells per gram (Figures 2A and 2B). The untreated tumor ranked in the upper quartile of 31 immunotherapy- and radiation-naive brain metastasis samples in our previously reported cohort.[10] The irradiated tumor was in the lower quintile of all brain metastases (Figures 2A and 2B) and contained the lowest $CD8^+$ T cell infiltration among our previously reported cohort of 14 lung metastases to the brain.[10] To identify radiation-associated changes in immune infiltrate in a comprehensive manner, we performed RNA sequencing, T cell receptor (TCR) sequencing, and cellular indexing of transcriptomes and epitopes (CITE) sequencing on single $CD45^+$ cells isolated from the untreated tumor, irradiated tumor, and pre-radiation and post-radiation PBMCs (Figure S2). Our CITE-seq panel included 137 antibodies specific for 130 distinct cell antigens.

Clustering on CITE-seq data identified thirteen broad $CD45^+$ cell types with varying distributions between tumor-infiltrating

CellPress
OPEN ACCESS

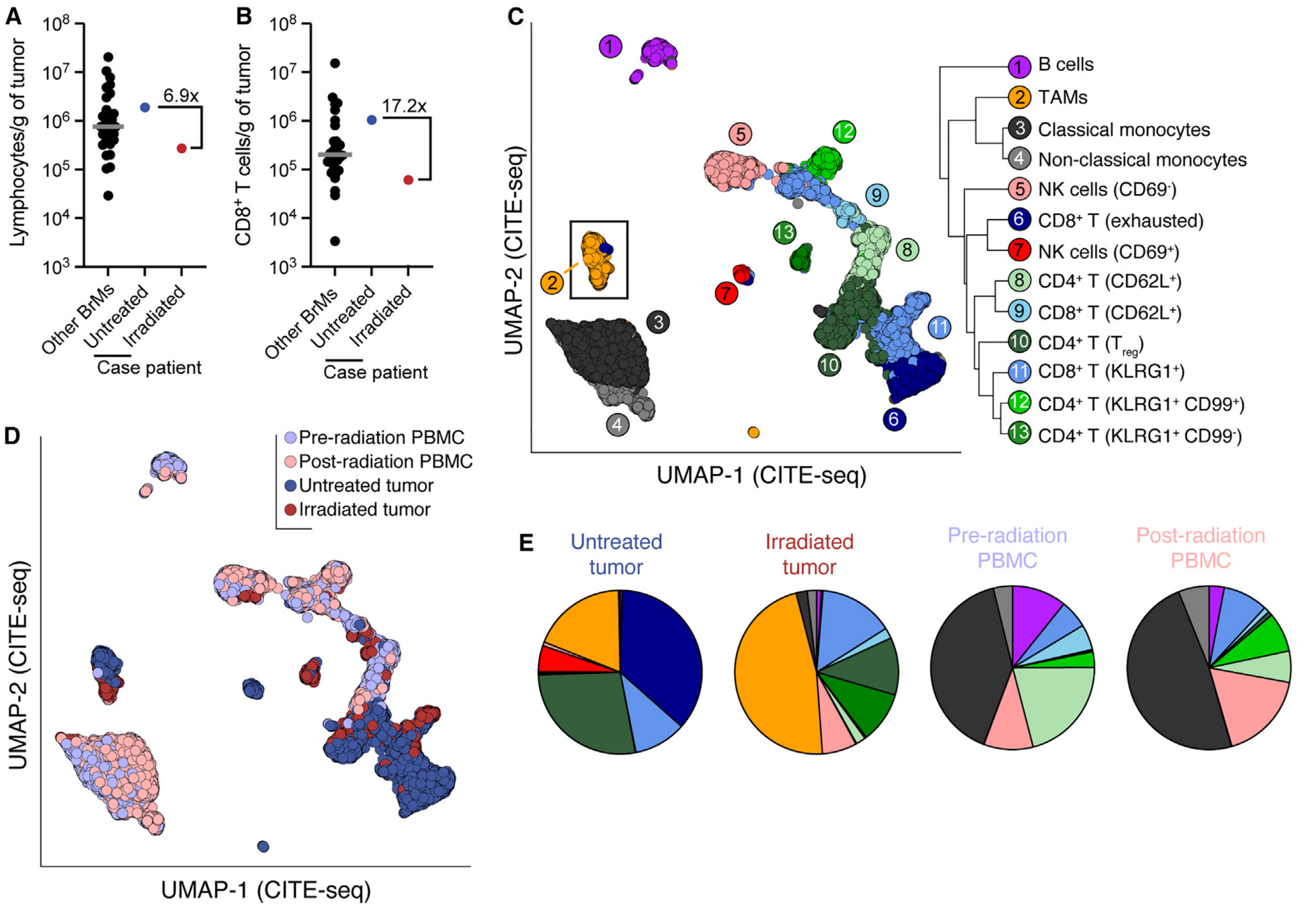

Figure 2. Reduced and altered immune cell infiltration in the irradiated tumor

(A and B) Flow cytometric quantification of total lymphocytic (A) and CD8$^+$ (B) T cell infiltration into the autologous untreated and irradiated tumors compared with brain metastases from 30 other individuals.[10] Gray bars indicate medians. BrMs, brain metastases.

(C) Uniform manifold approximation and projection (UMAP) and clustering of 45,947 cells sequenced from circulating and tumor-infiltrating CD45$^+$ cells. Protein expression data (CITE-seq) was used for clustering and projection.

(D) UMAP with cells colored by tissue of origin.

(E) Frequency of clusters among CD45$^+$ cells in each sample.

and circulating cells (Figures 2C–2E; Table S1). Notably, CD8$^+$ T cell frequency among CD45$^+$ cells was reduced from 47% in the untreated tumor to 17% in the irradiated tumor (Figure 2E), in line with flow cytometry results (55%–22%; Figures 2A and 2B). The frequency of tumor-associated macrophages (TAMs) among CD45$^+$ cells was 2.5-fold higher in the irradiated tumor (Figure 2E). In contrast to these drastic changes between the untreated and irradiated tumors, circulating CD45$^+$ subsets were broadly similar before and after treatment, indicating a localized—as opposed to systemic—remodeling of the anti-tumor immune response by radiation (Figure 2E).

Radiation-associated changes in tumor-infiltrating myeloid cells

Given the higher frequency of TAMs in the irradiated tumor, we performed a detailed analysis to determine phenotypic differences between the two tumor samples. Microglial markers *SALL1*, *TMEM119*, and *P2RY12* were minimally expressed among tumor-infiltrating cells, suggesting that microglia did not highly infiltrate the brain metastases in this patient (Figure S3). We performed subclustering of TAMs based on gene expression and found three subsets: TAM-1, TAM-2, and TAM-3 (Figure 3A; Table S2). TAM-1s expressed high protein levels of major histocompatibility complex (MHC) class II, CD40, and CD86, suggesting a role in promoting anti-tumor adaptive immune responses (Figure 3B). TAM-3s more closely resembled monocytes, with expression of *FCN1*, a marker of classical monocytes,[11] and *S100A12*, a gene expressed in classical monocytes and downregulated during monocyte-to-macrophage differentiation[12] (Figures 3C and 3D). TAM-2s appeared to be an intermediate population with shared characteristics with both the TAM-1 and TAM-3 subclusters.

While TAM-2s were present in both tumors, TAM-1s were predominately found in the untreated tumor and TAM-3s in the irradiated tumor (Figure 3E). These data are consistent with pro-inflammatory monocyte infiltration of tumors after radiation[13]

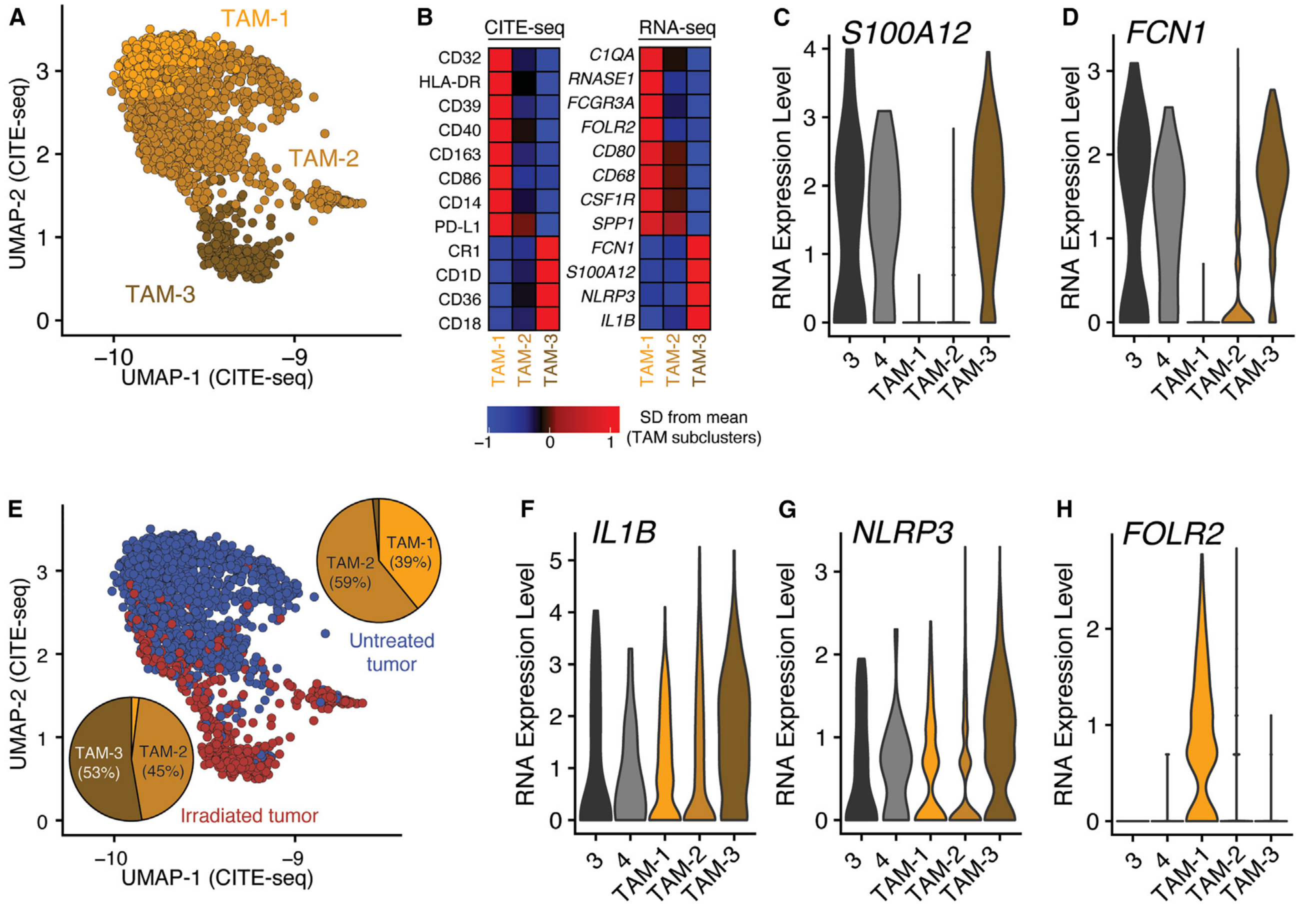

Figure 3. Distinct tumor-associated macrophage (TAM) populations infiltrate an irradiated and untreated brain metastasis

(A) UMAP projection of TAM subclusters (see inset in Figure 2C).

(B) Relative protein (left) and gene (right) expression of selected markers among TAM subclusters.

(C and D) Expression of monocyte markers *S100A12* and *FCN1* among monocyte clusters (3 and 4) and TAM subclusters (TAM-1, TAM-2, and TAM-3).

(E) UMAP projection of tumor-infiltrating TAMs, colored by tumor of origin. Pie charts show composition of TAMs in each tumor.

(F and G) Expression of monocyte activation-associated genes *IL1B*, which encodes IL-1β, and *NLRP3*, which encodes a key member of the inflammasome.

(H) Expression of *FOLR2*, which encodes the folate receptor β.

and also indicate a loss of tumor-resident MHC II^{+}, *FOLR2*$^{+}$ macrophages that efficiently prime effector CD8^{+} T cells and are associated with tumor control and patient survival[14,15] (Figures 3F–3H).

Radiation-associated phenotypic and clonotypic alterations in tumor-infiltrating T cells

T cell phenotype was also strikingly different between the irradiated and untreated tumors. In the untreated tumor, half of all T cells belonged to the exhausted CD8^{+} T cell cluster (Figures 2E, 4A, and 4B), characterized by expression of residence and checkpoint molecules such as PD-1, TIGIT, CTLA-4, LAG-3, CD69, and CD103 (Figure 4C). Exhausted CD8^{+} T cells were virtually absent in the irradiated tumor (Figure 4B), a striking contrast from our previous study of 31 radiation-naive brain metastases.[10] Overall, T cells in the irradiated tumor had a more memory- or progenitor-like phenotype compared with the untreated tumor, with higher expression of genes encoding TCF-1 (*TCF7*), CD127 (*IL7R*), and L-selectin/CD62L (*SELL*; Figure 4D).

The study of autologous samples allowed us to directly compare TCR sequences between the untreated and irradiated tumors. CD4^{+} and CD8^{+} T cells in the untreated tumor expressed a more clonal, extremely private TCR repertoire that was unshared with circulating T cells (Figures 4E–4H). Virtually no clonal overlap was detectable between the untreated tumor and circulating T cells from either time point (Figures 4F–4H). In contrast, high TCR overlap was present between the irradiated tumor and circulating T cells, particularly with those clones circulating at the time of the second surgical resection (Figures 4F–4H). This finding suggests near-total depletion of tumor-resident T cells by radiation and their subsequent replacement with circulating T cell clones. Given the exhausted phenotype of T cells in the untreated tumor and the low frequency of tumor-specific CD8^{+} T cells in the blood of patients with immunotherapy-naive cancer,[16,17] it is likely that most of

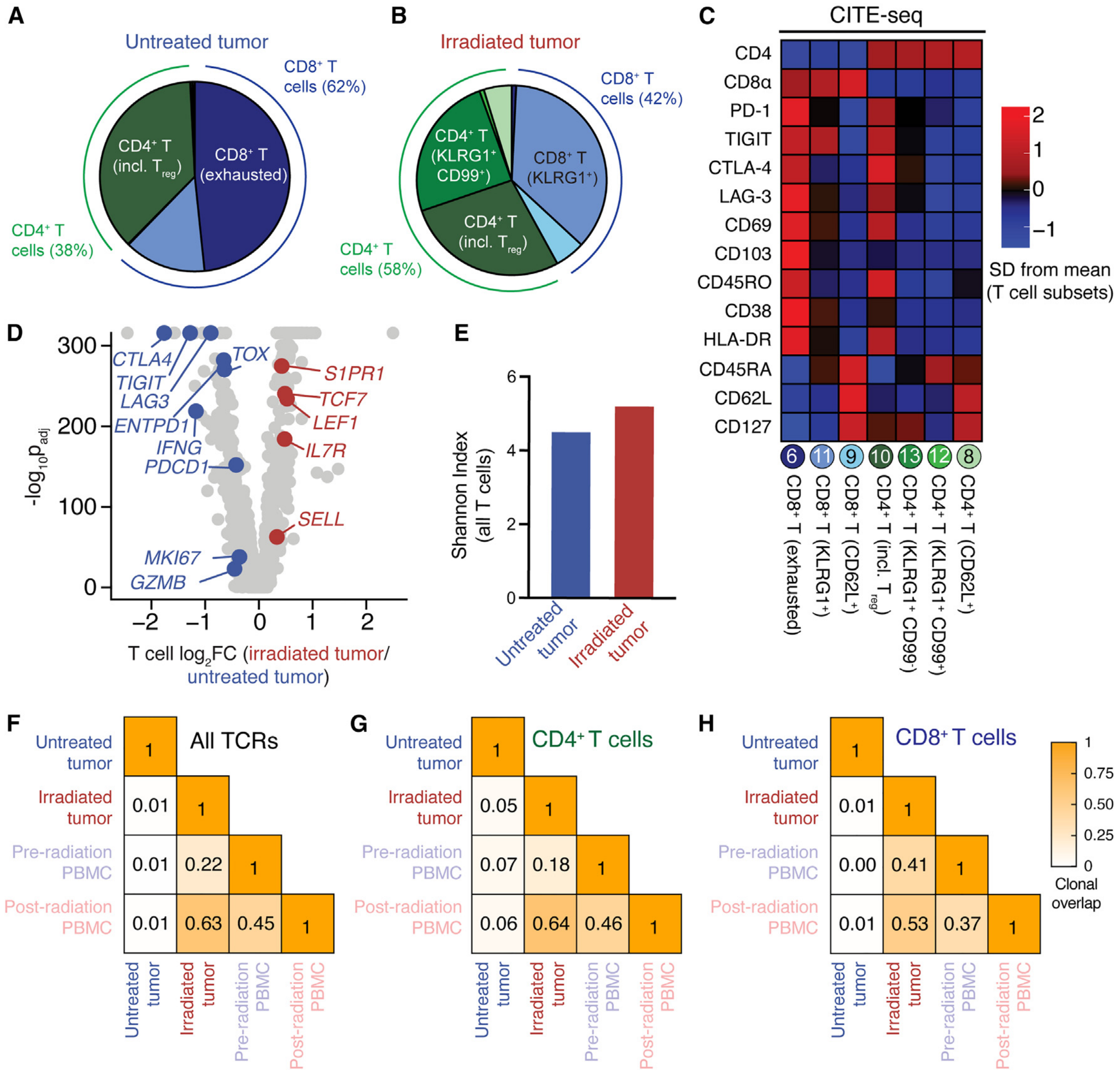

Figure 4. Dramatic differences in the phenotype and antigen specificity of tumor-infiltrating T cells in the untreated and irradiated tumors

(A and B) Frequency of each T cell cluster among tumor-infiltrating T cells in the (A) untreated and (B) irradiated tumors.

(C) Selected CITE-seq (protein) relative expression differences between T cell clusters.

(D) Volcano plot showing gene expression differences between total T cells from the irradiated and untreated tumors.

(E) Diversity of all T cell clones in the untreated and irradiated tumors as calculated by the Shannon diversity index.

(F–H) Clonal overlap (Morisita-Horn index) of all T cell receptor sequences between samples (F), those expressed in $CD4^+$ T cells (G), or in $CD8^+$ T cells (H). All T cells with a detected T cell receptor β (TCRβ) gene were used in TCR analyses.

these newly infiltrating T cells after radiation are not tumor specific. This interpretation is consistent with higher gene expression of effector- and division-associated genes (*GZMB*, *IFNG*, *MKI67*) in the untreated tumor and low expression of activation markers such as HLA-DR and CD38 in the irradiated tumor (Figures 4C and 4D). The lack of very early, TCR-driven activation markers such as CD69 and CD25 in the irradiated tumor also argues against nascent antigen-specific T cell activation in the untreated tumor (Figures S4A and S4B). Thus, despite their more functional phenotypic appearance (Figure 4D), $CD8^+$ T cells in the irradiated tumor may not contribute meaningfully to tumor-specific immunity.

Shared mutational and transcriptional profiles between the untreated and irradiated tumors

The striking differences in TCR repertoires between the untreated and irradiated tumors indicate the recognition of distinct sets of antigens by the infiltrating T cells in each tumor. To determine the somatic mutations and gene expression profiles of each tumor, we performed mRNA sequencing and whole-genome sequencing (WGS) on both tumors as well as PBMCs collected from each time point.

WGS was performed to depths of 167× and 179× on the untreated and irradiated tumors, respectively, and to 44× and 50× on the pre- and post-radiation PBMC samples, respectively. 475

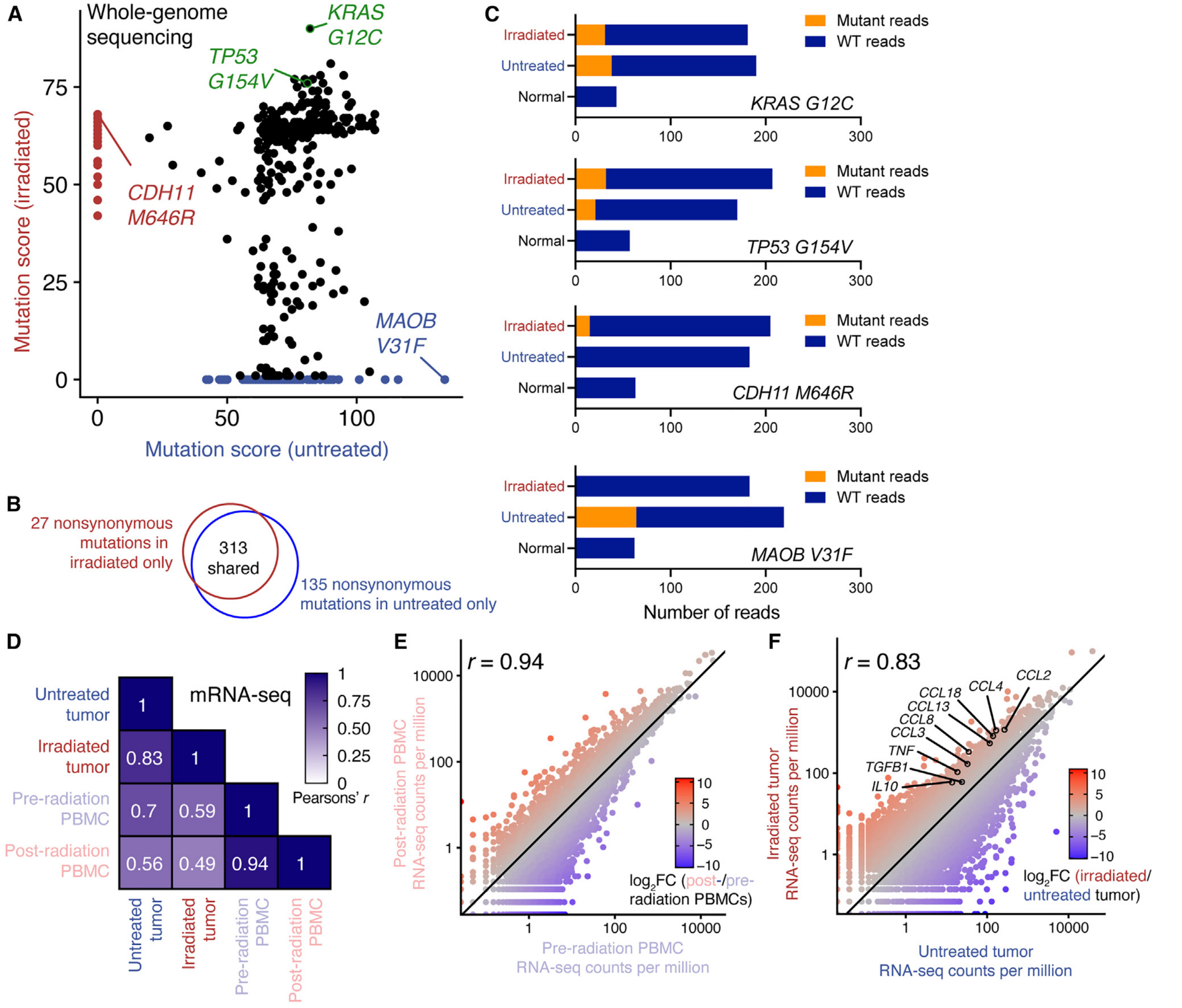

Figure 5. The untreated and irradiated tumors exhibit similar genetic and transcriptomic profiles

Whole-genome and mRNA sequencing were performed on both tumor samples.

(A) Mutation quality scores in the untreated and irradiated tumors for each of 475 non-synonymous single-nucleotide variants. A higher-quality score indicates a greater probability for the mutated allele to be present at a significantly different frequency in the tumor and normal (PBMC) samples.

(B) Venn diagram showing overlap in the two samples' non-synonymous mutational profiles.

(C) Example frequencies of shared (*KRAS* G12C, *TP53* G154V) and unshared (*CDH11* M646R, *MAOB* V31F) somatic mutations in the tumor and normal samples.

(D) Correlation of gene expression (measured by mRNA sequencing) between each sequenced sample.

(E) Correlation of gene expression between pre- and post-radiation PBMCs.

(F) Correlation of gene expression between the untreated and irradiated tumors. Selected cytokine genes are shown; see also Figure S5.

In (E) and (F), a slope of 1 is indicated with a solid line. mRNA was isolated from lymphocyte-depleted tumors (see STAR Methods).

single-nucleotide variations that caused non-synonymous changes in protein sequence were identified; 313 of these (66%) were shared between the tumors, including likely driver mutations *TP53* G115V and *KRAS* G12C (Figures 5A–5C). No *EGFR* mutations were detected in either tumor.

mRNA sequencing revealed a largely similar transcriptional profile (r = 0.94) between pre- and post-radiation PBMCs (Figures 5D and 5E). The untreated and irradiated tumors also exhibited broadly similar gene expression (r = 0.83; Figure 5F), but differences were evident in gene set enrichment analysis and cytokine gene expression (Figures 5F and S5; Table S3). Notably, human leukocyte antigen (HLA) gene expression, which encodes MHC molecules, was similar or slightly higher in the irradiated tumor, arguing against impaired antigen presentation as a basis for impaired T cell responses in the irradiated tumor (Figure S6). Further, monocyte chemoattractant protein-1 (*CCL2*) was highly

upregulated in the irradiated tumor, forming a potential basis for its high level of monocyte infiltration (Figure S6).

DISCUSSION

In this study, we report a detailed analysis of an irradiated and untreated brain metastasis from a single patient. This study has several important caveats, including its limited sample size, the potential confounding effects of pre-existing tumor heterogeneity, and the unusual clinical course of the patient. These are discussed in a separate section below. Nonetheless, these results raise important considerations for combined treatment with immunotherapy and radiation.

In our study, radiotherapy was associated with decreased immune infiltration between the two case patient lesions as well as when compared with 30 other patients with radiation- and immunotherapy-naive brain metastasis.[10] The ultimate outcome was a large, radiation-associated remodeling of tumor-infiltrating macrophages, T cells, and even natural killer (NK) cells (Figure 2E). The most notable difference in myeloid immune infiltrate between the two tumors was the higher frequency of monocyte-like cells in the irradiated tumor. These cells expressed high levels of monocyte marker genes found in circulating monocytes and also high levels of *IL1B*, indicating an activated phenotype. This infiltration of irradiated tumors by monocytes has been reported in pre-clinical models and is mediated by CCL2[13,18]; notably, we also find higher levels *CCL2* mRNA in the irradiated tumor. In pre-clinical models, CCL2 blockade in combination with radiation delayed tumor growth and decreased tumor proliferation and vascularity.[13,18] These results highlight the potential of TAM-modulating therapies targeting the CCL2/CCR2 axis or other pathways.[19,20]

The T cell infiltrate following radiation superficially appeared more phenotypically functional with a more diverse TCR repertoire (in line with previous studies[21]), lower expression of PD-1, and higher expression of TCF-1. However, we find that this is likely explained by infiltration of circulating T cells that are enriched for non-tumor-specific antigen reactivity and are unlikely to contribute to tumor-specific immunity.[16,17] Further, markers of nascent, antigen-specific activation such as CD69 and CD25 were not expressed among tumor-infiltrating T cells in the irradiated tumor, arguing against their priming in the irradiated tumor at this time interval after radiation.

Perhaps most importantly, we find that radiation depleted tumor-resident T cells; this has important implications for the sequencing of radiation therapy and checkpoint blockade. An early burst of proliferating circulating CD8$^+$ T cells is associated with clinical response to PD-1 pathway blockade; however, circulating activated CD8$^+$ T cells eventually return to baseline levels following the presumed migration of these activated cells to tumor or other inflamed sites.[22] Thus, administration of radiation after checkpoint blockade may deplete recently arrived effector cells generated by PD-1 blockade or other immunotherapies. This is consistent with previous results showing that patients with melanoma brain metastasis receiving SRS during or before ipilimumab demonstrated better overall survival and local control than those receiving SRS after ipilimumab.[23] Conversely, given our observed infiltration of circulating T cell clones into the irradiated tumor, administration of PD-1 pathway blockade with or immediately following radiation may improve tumor-specific infiltration into irradiated tumors. This latter hypothesis is consistent with studies demonstrating benefit of radiation concomitant with or prior to checkpoint blockade.[4,24–26]

Limitations of the study

Our study is subject to two key limitations. First, since our samples are from two separate tumors, we cannot completely distinguish between pre-existing tumor heterogeneity and the effects of radiation. Thus, it is possible that the different immune profiles of the two studied tumors reflect pre-existing differences in the size, location, or immunologic infiltrate of the respective tumors. However, we consider this unlikely for several reasons. First, our observation of monocyte infiltration and elevated *CCL2* expression in the irradiated tumor is consistent with pre-clinical models that exhibit CCL2-mediated recruitment of monocytes following radiation.[13,18] Second, we have previously shown that brain-metastasis-infiltrating T cells express a resident phenotype and a distinct TCR repertoire that is highly dissimilar from circulating clones,[10] consistent with studies from other tumor types.[16,17,27] Third, a previous study of multiple metastases resected from multiple patients showed high levels of TCR overlap between different tumors.[28] Finally, and perhaps most convincingly, the irradiated lesion is remarkable for its low infiltration and lack of exhausted CD8$^+$ T cells when compared with our larger cohort of previously reported brain metastasis samples.[10] Thus, while the observed differences could potentially predate the radiation therapy, the reduced T cell number and increased TCR overlap with peripheral blood in the treated tumor are consistent with radiation-induced changes.

Second, we only analyze two tumors from a single patient with an unusual clinical course. Local progression after SRS from brain metastases is uncommon,[29–31] particularly in the short time frame reported here,[32] and it is possible that the *TP53/KRAS* co-mutation in these tumors contributed to radioresistance.[33] Moreover, the optimal dose and fractionation for large brain metastases not amenable to single-fraction SRS has not been established.[34] Our institutional standard is 30 Gy in 5 fractions for lesions 3 cm and larger, but given the heterogeneous approaches to SRS dosing, our findings may not be broadly generalizable to all dose and fractionation schedules of SRS. Moreover, this study captures the TME at a single time point after radiation therapy. Further remodeling of the brain metastasis immune microenvironment with time after radiation is likely.

Additionally, given the analysis of a single patient, our results may or may not be applicable to a broader patient population. Larger studies of radiation-naive and irradiated brain metastases are underway.[35] However, even in studies with large numbers of patients, a lack of patient-matched samples and/or TCR profiling risks confounding intrinsic radioresistance of pre-existing tumor-infiltrating T cells with post-radiation infiltration of circulating cells. Our study including these advantages is consistent with a number of studies showing T cell death at doses much lower than those given here,[7,36–38] including a study of patient-matched biopsies finding ~90% decreases in infiltrating CD8$^+$ T cells following radiation of cervical cancer at doses of 20–30 Gy.[39] Thus, our data are consistent in supporting near-total depletion of brain-metastasis-infiltrating T cells at the treatment

dose, subject to the confounding effect of pre-existing tumor heterogeneity discussed above.

STAR★METHODS

Detailed methods are provided in the online version of this paper and include the following:

- KEY RESOURCES TABLE
- RESOURCE AVAILABILITY
  - Lead contact
  - Materials availability
  - Data and code availability
- EXPERIMENTAL MODEL AND SUBJECT DETAILS
  - Human patients
- METHOD DETAILS
  - Tissue and PBMC processing
  - Single cell sequencing
  - Single-cell sequencing alignment and quality control
  - Whole genome sequencing
  - mRNA-sequencing
- QUANTIFICATION AND STATISTICAL ANALYSIS
  - Single-cell analysis
  - mRNA-sequencing

SUPPLEMENTAL INFORMATION

Supplemental information can be found online at https://doi.org/10.1016/j.xcrm.2023.101054.

ACKNOWLEDGMENTS

We thank Pauline Hamon, Ewelina Sobierajska, Andreas Wieland, and Rafi Ahmed for helpful discussions and the Emory Yerkes Nonhuman Primate Genomics Core for assistance with single-cell and mRNA sequencing experiments. This research was supported by R00AI153736, P51OD011132, S10OD026799, and P50CA217691 from the National Institutes of Health (NIH), a Nell W. and William Simpson Elkin Fellowship from Emory University to L.J.S., and a first-time tenure-track award (RR220069) from the Cancer Prevention and Research Institute of Texas (CPRIT) to W.H.H. W.H.H. is a CPRIT Scholar in Cancer Research. The content is solely the responsibility of the authors and does not necessarily represent the official views of the NIH.

AUTHOR CONTRIBUTIONS

J.J.O. performed surgical resection of brain metastases. W.H.H. and L.J.S. designed the study, performed experiments, and interpreted data. W.H.H. prepared the manuscript, with editing from J.J.O. and L.J.S.

DECLARATION OF INTERESTS

The authors declare no competing interests.

Received: June 15, 2022
Revised: February 2, 2023
Accepted: April 27, 2023
Published: May 19, 2023

REFERENCES

1. Robert, C. (2020). A decade of immune-checkpoint inhibitors in cancer therapy. Nat. Commun. *11*, 3801. https://doi.org/10.1038/s41467-020-17670-y.
2. Callahan, M.K., Postow, M.A., and Wolchok, J.D. (2014). CTLA-4 and PD-1 pathway blockade: combinations in the clinic. Front. Oncol. *4*, 385. https://doi.org/10.3389/fonc.2014.00385.
3. Kalbasi, A., June, C.H., Haas, N., and Vapiwala, N. (2013). Radiation and immunotherapy: a synergistic combination. J. Clin. Invest. *123*, 2756–2763. https://doi.org/10.1172/jci69219.
4. Antonia, S.J., Villegas, A., Daniel, D., Vicente, D., Murakami, S., Hui, R., Yokoi, T., Chiappori, A., Lee, K.H., de Wit, M., et al. (2017). Durvalumab after chemoradiotherapy in stage III non–small-cell lung cancer. N. Engl. J. Med. *377*, 1919–1929. https://doi.org/10.1056/NEJMoa1709937.
5. Kelly, R.J., Ajani, J.A., Kuzdzal, J., Zander, T., Van Cutsem, E., Piessen, G., Mendez, G., Feliciano, J., Motoyama, S., Lièvre, A., et al. (2021). Adjuvant nivolumab in resected esophageal or gastroesophageal junction cancer. N. Engl. J. Med. *384*, 1191–1203. https://doi.org/10.1056/NEJMoa2032125.
6. Gough, M.J., and Crittenden, M.R. (2022). The paradox of radiation and T cells in tumors. Neoplasia *31*, 100808. https://doi.org/10.1016/j.neo.2022.100808.
7. Falcke, S.E., Rühle, P.F., Deloch, L., Fietkau, R., Frey, B., and Gaipl, U.S. (2018). Clinically relevant radiation exposure differentially impacts forms of cell death in human cells of the innate and adaptive immune system. Int. J. Mol. Sci. *19*, 3574. https://doi.org/10.3390/ijms19113574.
8. Nakamura, N., Kusunoki, Y., and Akiyama, M. (1990). Radiosensitivity of CD4 or CD8 positive human T-lymphocytes by an in vitro colony formation assay. Radiat. Res. *123*, 224–227. https://doi.org/10.2307/3577549.
9. Badiyan, S.N., Regine, W.F., and Mehta, M. (2016). Stereotactic radiosurgery for treatment of brain metastases. J. Oncol. Pract. *12*, 703–712. https://doi.org/10.1200/jop.2016.012922.
10. Sudmeier, L.J., Hoang, K.B., Nduom, E.K., Wieland, A., Neill, S.G., Schniederjan, M.J., Ramalingam, S.S., Olson, J.J., Ahmed, R., and Hudson, W.H. (2022). Distinct phenotypic states and spatial distribution of CD8+ T cell clonotypes in human brain metastases. Cell Rep. Med. *3*, 100620. https://doi.org/10.1016/j.xcrm.2022.100620.
11. Zilionis, R., Engblom, C., Pfirschke, C., Savova, V., Zemmour, D., Saatcioglu, H.D., Krishnan, I., Maroni, G., Meyerovitz, C.V., Kerwin, C.M., et al. (2019). Single-cell transcriptomics of human and mouse lung cancers reveals conserved myeloid populations across individuals and species. Immunity *50*, 1317–1334.e10. https://doi.org/10.1016/j.immuni.2019.03.009.
12. Martinez, F.O., Gordon, S., Locati, M., and Mantovani, A. (2006). Transcriptional profiling of the human monocyte-to-macrophage differentiation and polarization: new molecules and patterns of gene expression. J. Immunol. *177*, 7303–7311. https://doi.org/10.4049/jimmunol.177.10.7303.
13. Mondini, M., Loyher, P.-L., Hamon, P., Gerbé de Thoré, M., Laviron, M., Berthelot, K., Clémenson, C., Salomon, B.L., Combadière, C., Deutsch, E., and Boissonnas, A. (2019). CCR2-Dependent recruitment of Tregs and monocytes following radiotherapy is associated with TNFα-mediated resistance. Cancer Immunol. Res. *7*, 376–387. https://doi.org/10.1158/2326-6066.Cir-18-0633.
14. Nalio Ramos, R., Missolo-Koussou, Y., Gerber-Ferder, Y., Bromley, C.P., Bugatti, M., Núñez, N.G., Tosello Boari, J., Richer, W., Menger, L., Denizeau, J., et al. (2022). Tissue-resident FOLR2+ macrophages associate with CD8+ T cell infiltration in human breast cancer. Cell *185*, 1189–1207.e25. https://doi.org/10.1016/j.cell.2022.02.021.
15. Wang, B., Li, Q., Qin, L., Zhao, S., Wang, J., and Chen, X. (2011). Transition of tumor-associated macrophages from MHC class IIhi to MHC class IIlow mediates tumor progression in mice. BMC Immunol. *12*, 43. https://doi.org/10.1186/1471-2172-12-43.
16. Eberhardt, C.S., Kissick, H.T., Patel, M.R., Cardenas, M.A., Prokhnevska, N., Obeng, R.C., Nasti, T.H., Griffith, C.C., Im, S.J., Wang, X., et al. (2021). Functional HPV-specific PD-1+ stem-like CD8 T cells in head and neck cancer. Nature *597*, 279–284. https://doi.org/10.1038/s41586-021-03862-z.

CellPress
OPEN ACCESS

17. Oliveira, G., Stromhaug, K., Klaeger, S., Kula, T., Frederick, D.T., Le, P.M., Forman, J., Huang, T., Li, S., Zhang, W., et al. (2021). Phenotype, specificity and avidity of antitumour CD8+ T cells in melanoma. Nature *596*, 119–125. https://doi.org/10.1038/s41586-021-03704-y.

18. Kalbasi, A., Komar, C., Tooker, G.M., Liu, M., Lee, J.W., Gladney, W.L., Ben-Josef, E., and Beatty, G.L. (2017). Tumor-derived CCL2 mediates resistance to radiotherapy in pancreatic ductal adenocarcinoma. Clin. Cancer Res. *23*, 137–148. https://doi.org/10.1158/1078-0432.Ccr-16-0870.

19. Li, X., Yao, W., Yuan, Y., Chen, P., Li, B., Li, J., Chu, R., Song, H., Xie, D., Jiang, X., and Wang, H. (2017). Targeting of tumour-infiltrating macrophages via CCL2/CCR2 signalling as a therapeutic strategy against hepatocellular carcinoma. Gut *66*, 157–167. https://doi.org/10.1136/gutjnl-2015-310514.

20. Anfray, Ummarino, Andón, and Allavena. (2019). Current strategies to target tumor-associated-macrophages to improve anti-tumor immune responses. Cells *9*, 46. https://doi.org/10.3390/cells9010046.

21. Twyman-Saint Victor, C., Rech, A.J., Maity, A., Rengan, R., Pauken, K.E., Stelekati, E., Benci, J.L., Xu, B., Dada, H., Odorizzi, P.M., et al. (2015). Radiation and dual checkpoint blockade activate non-redundant immune mechanisms in cancer. Nature *520*, 373–377. https://doi.org/10.1038/nature14292.

22. Kamphorst, A.O., Pillai, R.N., Yang, S., Nasti, T.H., Akondy, R.S., Wieland, A., Sica, G.L., Yu, K., Koenig, L., Patel, N.T., et al. (2017). Proliferation of PD-1+ CD8 T cells in peripheral blood after PD-1–targeted therapy in lung cancer patients. Proc. Natl. Acad. Sci. USA *114*, 4993–4998. https://doi.org/10.1073/pnas.1705327114.

23. Kiess, A.P., Wolchok, J.D., Barker, C.A., Postow, M.A., Tabar, V., Huse, J.T., Chan, T.A., Yamada, Y., and Beal, K. (2015). Stereotactic radiosurgery for melanoma brain metastases in patients receiving ipilimumab: safety profile and efficacy of combined treatment. Int. J. Radiat. Oncol. Biol. Phys. *92*, 368–375. https://doi.org/10.1016/j.ijrobp.2015.01.004.

24. Qian, J.M., Yu, J.B., Kluger, H.M., and Chiang, V.L.S. (2016). Timing and type of immune checkpoint therapy affect the early radiographic response of melanoma brain metastases to stereotactic radiosurgery. Cancer *122*, 3051–3058. https://doi.org/10.1002/cncr.30138.

25. Shaverdian, N., Lisberg, A.E., Bornazyan, K., Veruttipong, D., Goldman, J.W., Formenti, S.C., Garon, E.B., and Lee, P. (2017). Previous radiotherapy and the clinical activity and toxicity of pembrolizumab in the treatment of non-small-cell lung cancer: a secondary analysis of the KEYNOTE-001 phase 1 trial. Lancet Oncol. *18*, 895–903. https://doi.org/10.1016/s1470-2045(17)30380-7.

26. Theelen, W.S.M.E., Chen, D., Verma, V., Hobbs, B.P., Peulen, H.M.U., Aerts, J.G.J.V., Bahce, I., Niemeijer, A.L.N., Chang, J.Y., de Groot, P.M., et al. (2021). Pembrolizumab with or without radiotherapy for metastatic non-small-cell lung cancer: a pooled analysis of two randomised trials. Lancet Respir. Med. *9*, 467–475. https://doi.org/10.1016/s2213-2600(20)30391-x.

27. Caushi, J.X., Zhang, J., Ji, Z., Vaghasia, A., Zhang, B., Hsiue, E.H.-C., Mog, B.J., Hou, W., Justesen, S., Blosser, R., et al. (2021). Transcriptional programs of neoantigen-specific TIL in anti-PD-1-treated lung cancers. Nature *596*, 126–132. https://doi.org/10.1038/s41586-021-03752-4.

28. Kalaora, S., Wolf, Y., Feferman, T., Barnea, E., Greenstein, E., Reshef, D., Tirosh, I., Reuben, A., Patkar, S., Levy, R., et al. (2018). Combined analysis of antigen presentation and T-cell recognition reveals restricted immune responses in melanoma. Cancer Discov. *8*, 1366–1375. https://doi.org/10.1158/2159-8290.Cd-17-1418.

29. Gui, C., Grimm, J., Kleinberg, L.R., Zaki, P., Spoleti, N., Mukherjee, D., Bettegowda, C., Lim, M., and Redmond, K.J. (2020). A dose-response model of local tumor control probability after stereotactic radiosurgery for brain metastases resection cavities. Adv. Radiat. Oncol. *5*, 840–849. https://doi.org/10.1016/j.adro.2020.06.007.

30. de Azevedo Santos, T.R., Tundisi, C.F., Ramos, H., Maia, M.A.C., Pellizzon, A.C.A., Silva, M.L.G., Fogaroli, R.C., Chen, M.J., Suzuki, S.H., Dias, J.E.S., Jr., et al. (2015). Local control after radiosurgery for brain metastases: predictive factors and implications for clinical decision. Radiat. Oncol. *10*, 63. https://doi.org/10.1186/s13014-015-0367-y.

31. Vogelbaum, M.A., Angelov, L., Lee, S.-Y., Li, L., Barnett, G.H., and Suh, J.H. (2006). Local control of brain metastases by stereotactic radiosurgery in relation to dose to the tumor margin. J. Neurosurg. *104*, 907–912. https://doi.org/10.3171/jns.2006.104.6.907.

32. Churilla, T.M., Chowdhury, I.H., Handorf, E., Collette, L., Collette, S., Dong, Y., Alexander, B.M., Kocher, M., Soffietti, R., Claus, E.B., and Weiss, S.E. (2019). Comparison of local control of brain metastases with stereotactic radiosurgery vs surgical resection. JAMA Oncol. *5*, 243–247. https://doi.org/10.1001/jamaoncol.2018.4610.

33. Hong, T.S., Wo, J.Y., Borger, D.R., Yeap, B.Y., McDonnell, E.I., Willers, H., Blaszkowsky, L.S., Kwak, E.L., Allen, J.N., Clark, J.W., et al. (2017). Phase II study of proton-based stereotactic body radiation therapy for liver metastases: importance of tumor genotype. J. Natl. Cancer Inst. *109*. https://doi.org/10.1093/jnci/djx031.

34. Masucci, G.L. (2018). Hypofractionated radiation therapy for large brain metastases. Front. Oncol. *8*, 379. https://doi.org/10.3389/fonc.2018.00379.

35. Buchwald, Z., Jansen, C., Prabhu, R., Pagadala, M., Chappa, P., Goyal, S., Zhou, C., Neill, S., Prokhnevska, N., Cardenas, M., et al. (2023). Immune niches in brain metastases contain TCF1+ stem-like T cells, are associated with disease control and are modulated by preoperative SRS. Res. Sq. https://doi.org/10.21203/rs.3.rs-2722744/v1.

36. Heylmann, D., Ponath, V., Kindler, T., and Kaina, B. (2021). Comparison of DNA repair and radiosensitivity of different blood cell populations. Sci. Rep. *11*, 2478. https://doi.org/10.1038/s41598-021-81058-1.

37. Pugh, J.L., Sukhina, A.S., Seed, T.M., Manley, N.R., Sempowski, G.D., van den Brink, M.R.M., Smithey, M.J., and Nikolich-Žugich, J. (2014). Histone deacetylation critically determines T cell subset radiosensitivity. J. Immunol. *193*, 1451–1458. https://doi.org/10.4049/jimmunol.1400434.

38. Nakamura, N., Kusunoki, Y., and Akiyama, M. (1990). Radiosensitivity of CD4 or CD8 positive human T-lymphocytes by an in vitro colony formation assay. Radiat. Res. *123*, 224–227.

39. Qinfeng, S., Depu, W., Xiaofeng, Y., Shah, W., Hongwei, C., and Yili, W. (2013). In situ observation of the effects of local irradiation on cytotoxic and regulatory T lymphocytes in cervical cancer tissue. Radiat. Res. *179*, 584–589. https://doi.org/10.1667/rr3155.1.

40. Hao, Y., Hao, S., Andersen-Nissen, E., Mauck, W.M., Zheng, S., Butler, A., Lee, M.J., Wilk, A.J., Darby, C., Zager, M., et al. (2021). Integrated analysis of multimodal single-cell data. Cell *184*, 3573–3587.e29. https://doi.org/10.1016/j.cell.2021.04.048.

41. Li, H., and Durbin, R. (2009). Fast and accurate short read alignment with Burrows-Wheeler transform. Bioinformatics *25*, 1754–1760. https://doi.org/10.1093/bioinformatics/btp324.

42. Dobin, A., Davis, C.A., Schlesinger, F., Drenkow, J., Zaleski, C., Jha, S., Batut, P., Chaisson, M., and Gingeras, T.R. (2013). STAR: ultrafast universal RNA-seq aligner. Bioinformatics *29*, 15–21. https://doi.org/10.1093/bioinformatics/bts635.

43. Robinson, M.D., McCarthy, D.J., and Smyth, G.K. (2010). edgeR: a Bioconductor package for differential expression analysis of digital gene expression data. Bioinformatics *26*, 139–140. https://doi.org/10.1093/bioinformatics/btp616.

44. Kim, S., Scheffler, K., Halpern, A.L., Bekritsky, M.A., Noh, E., Källberg, M., Chen, X., Kim, Y., Beyter, D., Krusche, P., and Saunders, C.T. (2018). Strelka2: fast and accurate calling of germline and somatic variants. Nat. Methods *15*, 591–594. https://doi.org/10.1038/s41592-018-0051-x.

45. Chen, X., Schulz-Trieglaff, O., Shaw, R., Barnes, B., Schlesinger, F., Källberg, M., Cox, A.J., Kruglyak, S., and Saunders, C.T. (2016). Manta: rapid detection of structural variants and indels for germline and cancer sequencing applications. Bioinformatics *32*, 1220–1222. https://doi.org/10.1093/bioinformatics/btv710.

46. Wang, K., Li, M., and Hakonarson, H. (2010). ANNOVAR: functional annotation of genetic variants from high-throughput sequencing data. Nucleic Acids Res. *38*, e164. https://doi.org/10.1093/nar/gkq603.
47. McInnes, L., Healy, J., Saul, N., and Großberger, L. (2018). UMAP: uniform manifold approximation and projection. J. Open Source Softw. *3*, 861. https://doi.org/10.21105/joss.00861.
48. Oksanen, J., Blanchet, F.G., Friendly, M., Kindt, R., Legendre, P., McGlinn, D., Minchin, P.R., O'Hara, R.B., Simpson, G.L., Solymos, P., et al. (2020). vegan: community ecology package. https://CRAN.R-project.org/package=vegan.
49. Wickham, H. (2016). ggplot2 : elegant graphics for data analysis. Use R!. https://doi.org/10.1007/978-3-319-24277-4.
50. Korotkevich, G., Sukhov, V., Budin, N., Shpak, B., Artyomov, M.N., and Sergushichev, A. (2021). Fast gene set enrichment analysis. Preprint at bioRxiv. https://doi.org/10.1101/060012.

CellPress
OPEN ACCESS

STAR★METHODS

KEY RESOURCES TABLE

REAGENT or RESOURCE	SOURCE	IDENTIFIER
Antibodies		
TotalSeq-C Human Universal Cocktail, V1.0	Biolegend	Cat# 399905; RRID# AB_2876728
Anti-CD45 PE	Miltenyi	Cat# 130-117-281
Anti-CD4 APC	Miltenyi	Cat# 130-113-222
Anti-CD8α BV510	Biolegend	Cat# 301048; RRID #AB_2561942
Chemicals, peptides, and recombinant proteins		
Zombie NIR Fixable Viability dye	Biolegend	Cat# 423106
Percoll	Sigma	Cat# P1644
CountBright Absolute Counting Beads	Fisher	C36950
Lymphocyte separation medium	Corning	Cat# 25-072-CV
Deposited data		
Pre-processed whole-genome and single-cell sequencing	This paper	Mendeley Data: https://doi.org/10.17632/kdmdc8pkjt.1
Software and algorithms		
Cell Ranger, version 6	10x Genomics	https://support.10xgenomics.com/single-cell-gene-expression/software/pipelines/latest/what-is-cell-ranger
Seurat	Hao et al.[40]	CRAN
FlowJo	BDflow	https://www.flowjo.com/
Burrows-Wheeler Aligner	Li & Durbin[41]	https://github.com/lh3/bwa
Manta	Illumina	https://github.com/Illumina/manta
Strelka	Illumina	https://github.com/Illumina/strelka
STAR Aligner	Dobin et al.[42]	https://github.com/alexdobin/STAR
edgeR	Robinson et al.[43]	Bioconductor
ggplot2	https://ggplot2.tidyverse.org	CRAN

RESOURCE AVAILABILITY

Lead contact

Further information and requests for resources and reagents should be directed to and will be fulfilled by the lead contact, Lisa Sudmeier (lisa.jane.sudmeier@emory.edu).

Materials availability

This study did not generate new reagents.

Data and code availability

- The raw sequencing data cannot be deposited in a public repository due to privacy concerns. Original sequencing data is available upon request to the lead contact and with permission of the Emory University Institutional Review Board. Pre-processed whole-genome and single-cell sequencing data have instead been deposited at Mendeley Data and are publicly available as of the date of publication. Accession numbers are listed in the key resources table.
- This paper does not report original code. Published software packages used in this study are listed in the key resources table.
- Any additional information required to reanalyze the data reported in this paper is available from the lead contact upon request.

EXPERIMENTAL MODEL AND SUBJECT DETAILS

Human patients

Tumor tissue and peripheral blood were collected from immunotherapy-naïve patients undergoing resection of brain metastases at Emory University Hospital. Informed consent was obtained, with experiments carried out with the approval of the Emory University

Institutional Review Board under protocols IRB00045732, IRB00095411, and STUDY00001995. All consenting patients were included in sample collection unless insufficient tissue was available. We recently published[10] a detailed characterization of immune infiltrate in brain metastases from this cohort (Sudmeier et al., *Cell Reports Medicine* 2022). The samples in this study were collected as part of ongoing sample collection as described in Sudmeier et al.; however, we did not report data from the irradiated metastasis and matched PBMCs in that study.

Blood was collected in lithium heparin tubes at the time of surgery, and blood and tumor tissue were stored at 4°C until samples were processed, typically in less than 1 h. The patient was immunotherapy-naïve but received dexamethasone (10 mg bolus +4 mg/6 h) two days prior to the first surgery and three days prior to the second surgery.

METHOD DETAILS

Tissue and PBMC processing

Four samples were studied here (Figure 1A): an untreated tumor, an irradiated tumor, and matched PBMCs from the time of each resection ("pre-radiation PBMCs" and "post-radiation PBMCs"). Brain metastasis tissues and matched PBMCs were collected at the time of surgery and processed as previously described.[10] Briefly, tumors were weighed, cut into pieces, and digested with an enzymatic cocktail for 1 h at 37°C. A single-cell suspension was generated using a cell strainer, and cells were pelleted and washed in PBS with 2% fetal bovine serum (FBS). A 44%/67% Percoll gradient was used to separate white blood cells, and the interface was collected and washed following centrifugation of the gradient. PBMCs were isolated by underlaying whole blood with Corning Lymphocyte Separation Medium and centrifuging for 20 min. The interface was collected and washed in PBS with 2% FBS.

To quantify immune infiltrate, a small volume of freshly-isolated tumor-infiltrating cells and PBMCs were incubated with CountBright counting beads, stained with fluorescently-labeled anti-CD45 and anti-CD8 antibodies, and analyzed on a BD LSR II cytometer to determine absolute numbers of lymphocytes and $CD8^+$ cells. The remainder of cells were resuspended in FBS with 10% DMSO, frozen to −80 °C at a cooling rate of 1°C/min, and stored in liquid nitrogen until use in single-cell sequencing experiments.

Single cell sequencing

Aliquots of cryopreserved cells from each sample were simultaneously and rapidly thawed, washed twice in RPMI with 10% FBS, and counted. Cells were stained with Zombie NIR viability dye (BioLegend), anti-CD45 PE, anti-CD4 APC, anti-CD8α BV510, and the TotalSeq-C Universal Cocktail v1.0 (BioLegend) in PBS with 2% FBS and 2 mM EDTA. To avoid competition of fluorescent antibodies with CITE-sequencing antibodies, non-competing clones were used. For CD8α, clone RPA-T8 was used for fluorescent labeling and SK1 for CITE-seq; for CD45, clone REA1023 was used for fluorescent labeling and HI30 for CITE-seq; for CD4, clone REA623 for fluorescent labeling and RPA-T4 for CITE-seq. The TotalSeq-C Universal Cocktail v1.0 was added at 1 test per 6 x 10^5 cells. After staining, cells were washed in PBS with 2% FBS and 2 mM EDTA.

Following staining, cells were sorted on a BD FACS Aria II into RPMI with 10% FBS. We sorted live, $CD45^+$ cells from each sample. To further characterize T cell responses in the tumors, we also sorted $CD45^+$ cells positive for CD4 or CD8 for separate single-cell sequencing captures. This resulted in six total captures performed on a 10x Genomics Chromium controller in the Emory Yerkes Nonhuman Primate (NHP) Genomics Core: one capture of $CD45^+$ cells from each PBMC and tumor sample, and one capture each of $CD45^+$ cells positive for CD4 or CD8 from each tumor sample. Gene expression, CITE-sequencing, and T cell receptor (TCR) libraries were generated from each sample by the Emory Yerkes NHP Genomics Core. An overview of this experiment is shown in Figure S2A.

Single-cell sequencing alignment and quality control

Single-cell gene expression and CITE-seq data were aligned and mapped with Cell Ranger version 6 (10x Genomics). T cell receptor sequences were called with cellranger vdj. As described previously,[10] TCRβ sequences were used to identify T cell clonotypes. T cells sharing the same CDR3 amino acid sequence and using the same *TRBV* and *TRBJ* gene family were considered to belong to the same clonotype. 61,823 cells were sequenced. To exclude dead, doublet, and poorly-sequenced cells, cells with <0.8% or >10% of reads originating from mitochondrial genes, <800 total genes detected or <2,500 or >20,000 number of RNA molecules sequenced were excluded from further analysis. 45,947 cells met these quality control criteria. Final quality control data are shown in Figure S2B.

Whole genome sequencing

For whole genome sequencing (WGS), normal DNA was isolated from PBMCs with a Qiagen AllPrep kit using the manufacturer's instructions. Tumor DNA was isolated in an identical manner from the lymphocyte-depleted fraction of the tumor after the Percoll gradient. WGS was performed by Novogene. Normal samples were sequenced to a depth of 43.8x and 49.5x at the first and second timepoint, respectively. Tumor samples were sequenced to 166.5x and 178.7x depth for the untreated and irradiated tumor, respectively.

Sequencing reads were aligned with the Burrows-Wheeler Aligner.[41] Manta and Strelka were used to call indels and single nucleotide variants relative to pre-radiation PBMCs.[44,45] Annovar was used to annotate output.[46]

mRNA-sequencing

For mRNA-sequencing, RNA was isolated with the Qiagen AllPrep kit simultaneously with DNA isolation as described above. RNA was submitted to the Emory Yerkes NHP Genomics Core for paired-end sequencing. Reads were aligned to the human genome (GRCh38) and assigned to genes with the STAR aligner.[42] Count data were analyzed in R with the edgeR package.[43]

QUANTIFICATION AND STATISTICAL ANALYSIS

Single-cell analysis

Gene expression and antibody capture (CITE-seq) data were analyzed with Seurat v4.[40] Gene expression/RNA-sequencing data were normalized and scaled with the SCTransform command. Antibody capture data were normalized using the centered log ratio (CLR) transformation and subsequently scaled. UMAP projection[47] and clustering were performed with antibody capture data after removing isotype controls. 11 principal components, 200 neighbors, and a minimum distance of 0.1 were used for nearest-neighbor graph construction, clustering (resolution = 0.4), and UMAP projection.

For calculation of cluster frequencies among tumor-infiltrating or circulating $CD45^+$ cells, only $CD45^+$ captures were used (Figure 2E). TAMs were subclustered using the FindSubCluster command in Seurat with a resolution of 0.4 and using the original Louvain algorithm for modularity optimization. Differential gene expression was calculated in all cases using the FindMarkers command in Seurat. TCR diversity was quantified with the Shannon index calculated by the diversity command in the vegan package.[48] TCR overlap was quantified by the Morisita-Horn index. Plots were made with Seurat or ggplot2.[49]

mRNA-sequencing

Count data were normalized to counts per million with the cpm function in edgeR. Given the availability of only one sample per condition, differential expression analysis between the two tumor samples was performed using the exactTest function and tagwise dispersion from the PBMC samples. Gene set enrichment analysis was performed with the fgsea package using $\log_{10}$FDR*sign(fold change) as the ranking statistic.[50] Results – particularly p values – should be interpreted with caution given the availability of one tumor in each group.

Cell Reports Medicine, Volume *4*

Supplemental information

Immune microenvironment remodeling after radiation of a progressing brain metastasis

William H. Hudson, Jeffrey J. Olson, and Lisa J. Sudmeier

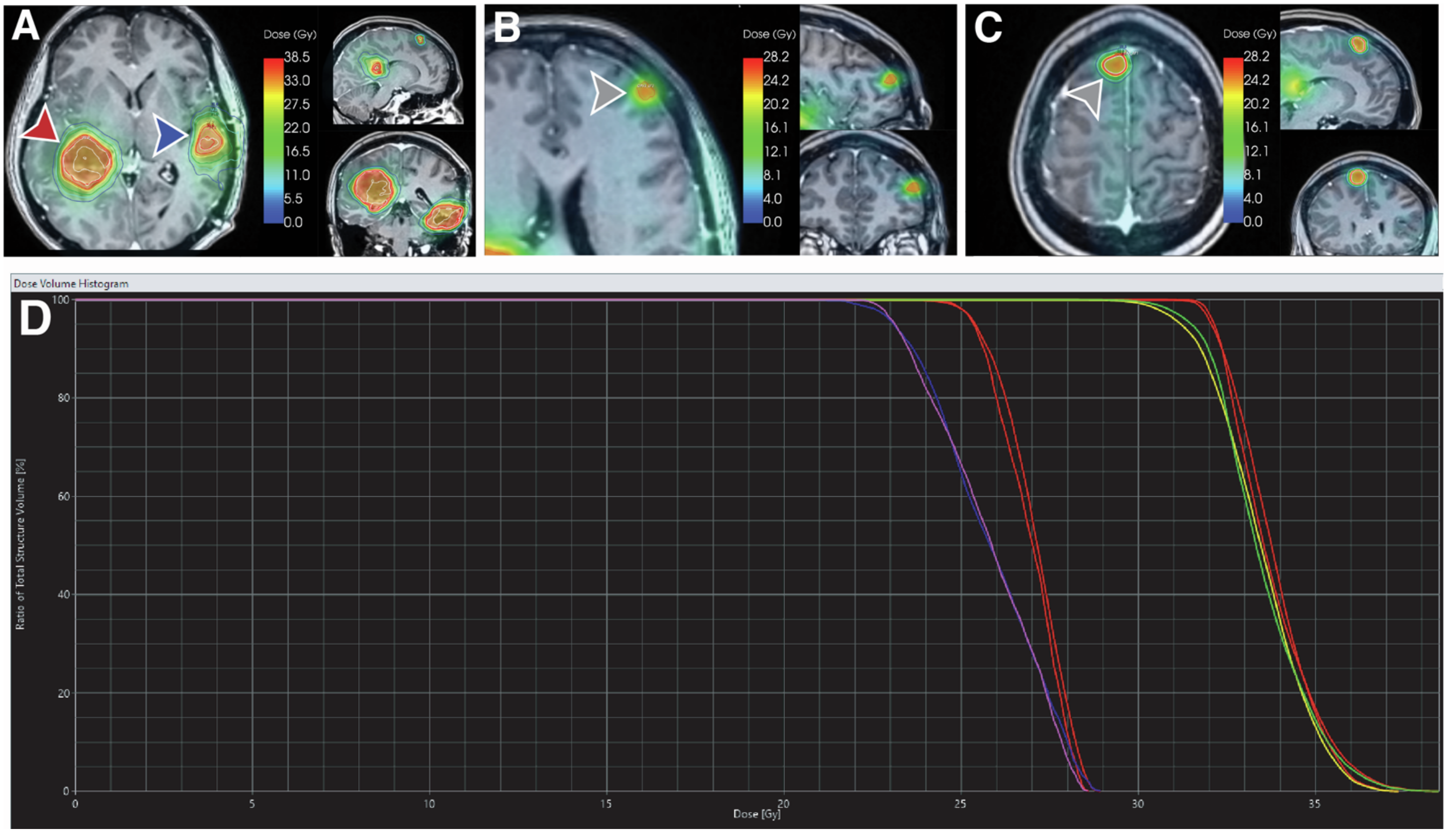

Figure S1, related to Figure 1: Stereotactic radiosurgery plans for the 4 treated lesions. **A)** The left temporal resection cavity (blue) and right frontoparietal lesion (red) that ultimately progressed were each treated to 30 Gy in 5 fractions of 6 Gy each. The unresected left frontal **(B)** and right frontal **(C)** tumors were each treated to 21 Gy in a single fraction. **D)** Dose volume showing coverage of the targets. Red lines show gross tumor volumes (GTVs) for each tumor. The GTV encompasses the entire contrast-enhancing tumor visualized on T1 post-contrast MRI. Red lines: Gross Tumor Volumes (GTV) for each tumor. Blue, Purple, Yellow, and Green lines: Planning Target Volume (PTV) for each tumor. The PTVs were generated by expanding the GTV 1mm isometrically, which the exception of the Left temporal lobe resection cavity GTV, which was expanded by 2mm to generate the PTV. Blue lines: Left Frontal PTV (shown in B); purple lines: Right Frontal PTV (shown in C); yellow lines: Left temporal resection cavity PTV (shown in A); green lines: right frontoparietal PTV (also shown in A)

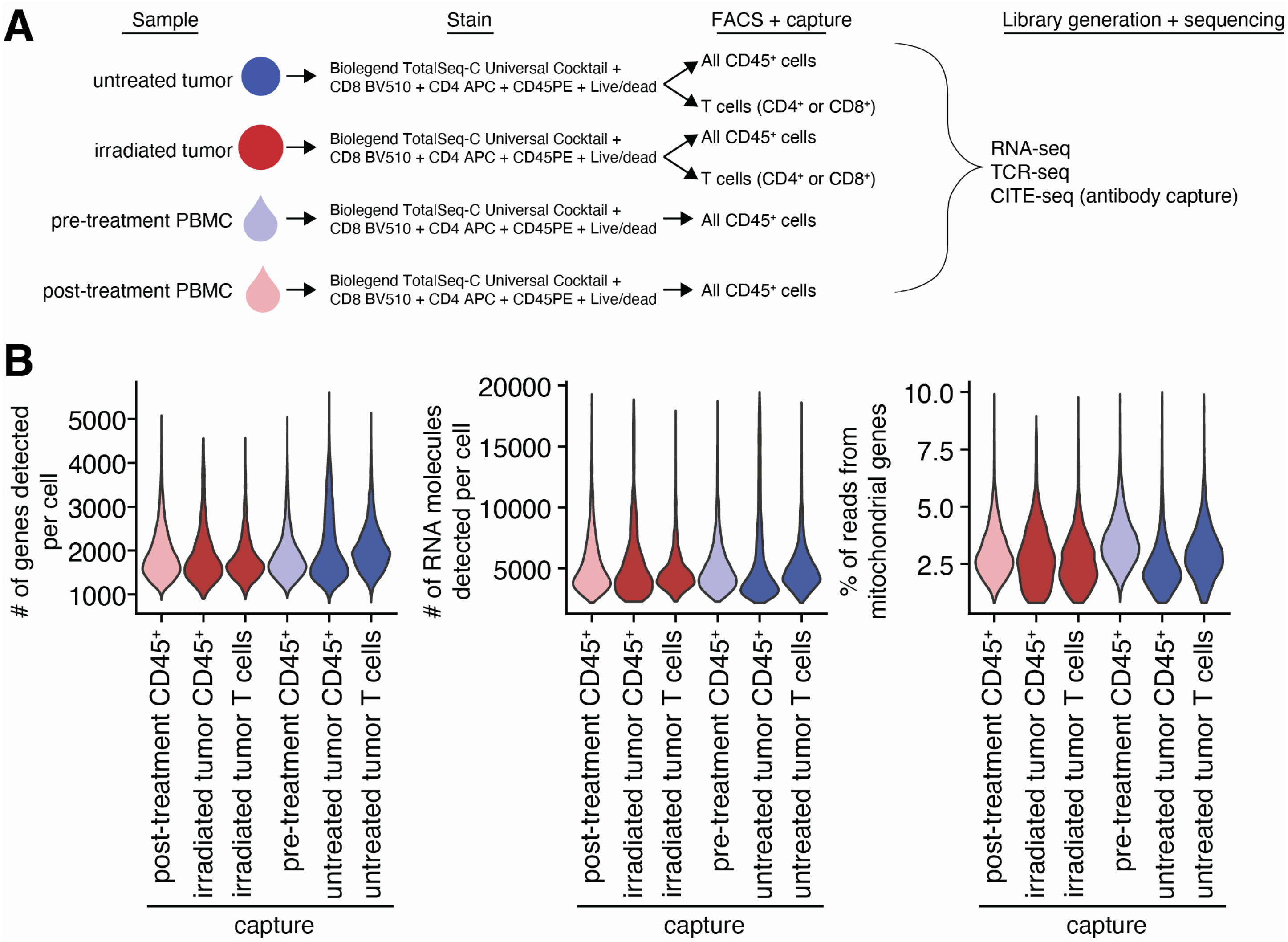

Figure S2, related to Figure 2: Single-cell experiments performed on samples described in this study. A) All four samples were stained with the Biolegend Totalseq-C Universal Cocktail, a mixture of antibodies against 130 unique surface antigens. Cells were additionally stained with fluorescently-labeled, non-competing clones against CD4, CD8, and CD45 for flow sorting. Live CD45^{+} cells were sorted from all samples, and an additional pool of T cells (CD45^{+} and CD4^{+} or CD8^{+}) were sorted from the two tumor samples. RNA-sequencing, TCR-sequencing, and CITE-sequencing (antibody capture) libraries were generated and sequenced from all sorted populations. **B)** Quality control metrics of gene expression libraries from all six samples.

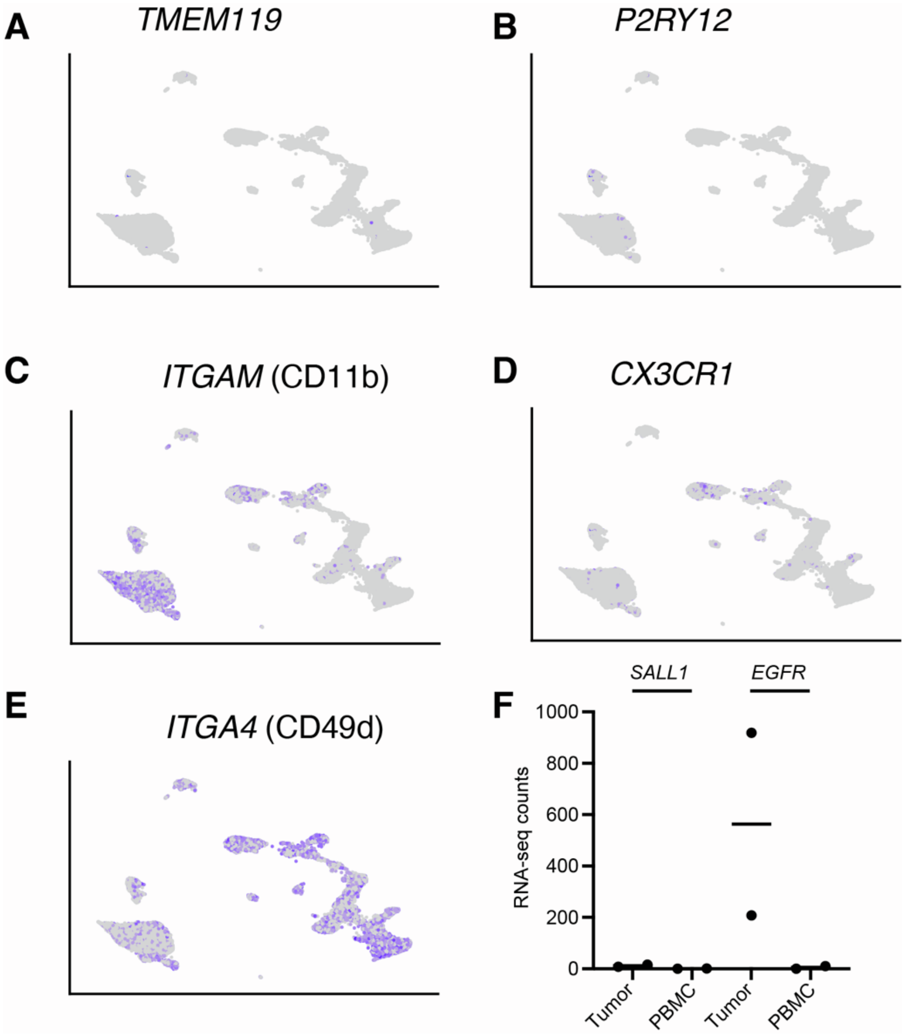

Figure S3, related to Figure 3: Limited expression of microglia-associated genes within the tumor. A-E) Expression of selected myeloid markers in single-cell RNA-sequencing data set. A and B indicate limited expression of microglia markers *TMEM119* and *P2RY12* genes among tumor-infiltrating immune cells. F) The microglia marker gene *SALL1* was not detected in single-cell RNA-sequencing data, and bulk RNA-sequencing data is shown instead. The lung tumor tissue marker gene *EGFR* is shown for comparison.

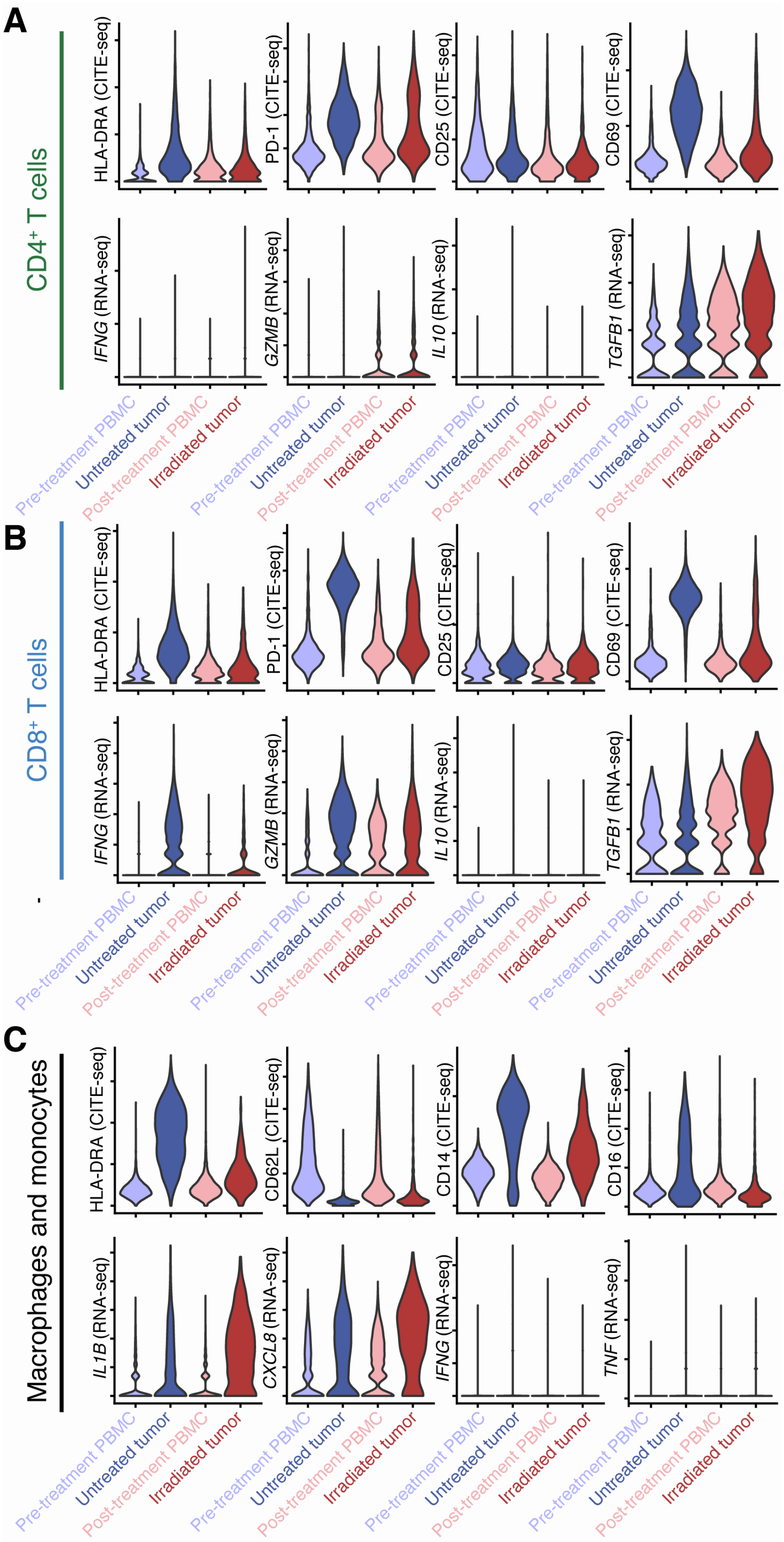

Figure S4, related to Figure 4: Expression of selected markers associated with activation and functional status of CD4^{+} T cells (A), CD8^{+} T cells (B), and macrophages/monocytes (C).

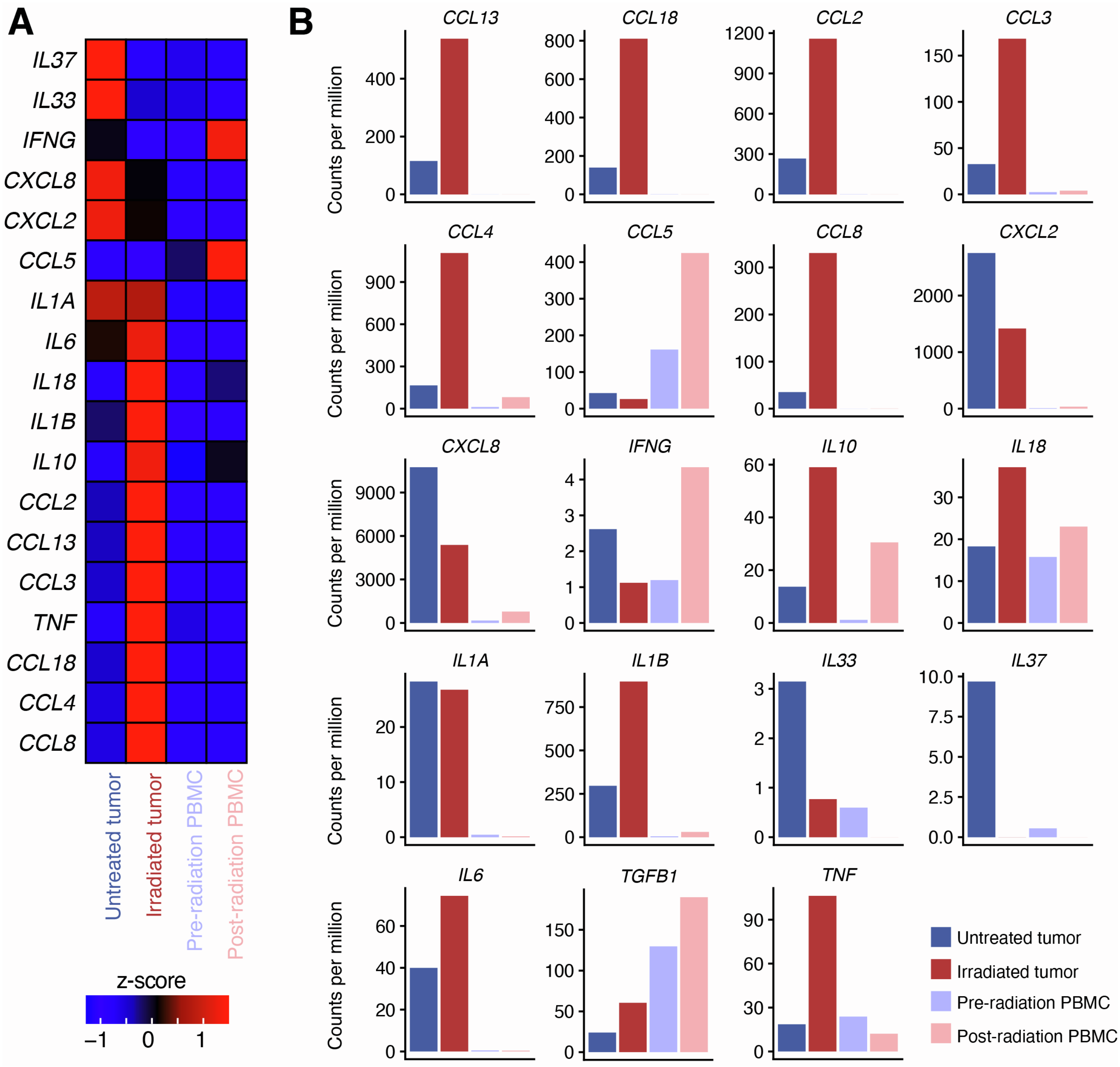

Figure S5, related to Figure 5: Relative (A) and absolute (B) expression of cytokine genes in PBMC and lymphocyte-depleted tumor samples.

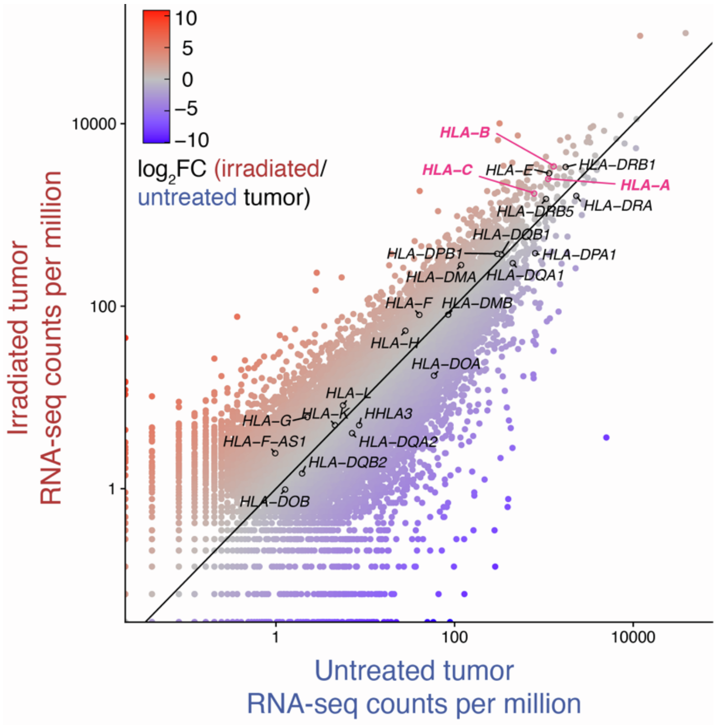

Figure S6, related to Figure 5: mRNA expression of HLA genes in the untreated (x-axis) and irradiated tumor (y-axis). HLA transcript expression was well correlated (r=0.84). Classical MHC I genes (HLA-A, -B, and -C) were among the highest expressed genes in both tumors and are shown labeled in pink.
